# Mechanistic Target of Rapamycin Complex 2 Regulation of the Primary Human Trophoblast Cell Transcriptome

**DOI:** 10.3389/fcell.2021.670980

**Published:** 2021-11-04

**Authors:** Fredrick J. Rosario, Amy Catherine Kelly, Madhulika B. Gupta, Theresa L. Powell, Laura Cox, Thomas Jansson

**Affiliations:** ^1^Division of Reproductive Sciences, Department of OB/GYN University of Colorado Anschutz Medical Campus, Aurora, CO, United States; ^2^Children’s Health Research Institute and Department of Pediatrics and Biochemistry, University of Western Ontario, London, ON, Canada; ^3^Section of Neonatology, Department of Pediatrics, University of Colorado Anschutz Medical Campus, Aurora, CO, United States; ^4^Section of Molecular Medicine, Department of Internal Medicine, Center for Precision Medicine, Wake Forest School of Medicine, Winston-Salem, NC, United States

**Keywords:** placenta, maternal-fetal exchange, human, nutrient sensor, gene array

## Abstract

Mechanistic Target of Rapamycin Complex 2 (mTORC2) regulates placental amino acid and folate transport. However, the role of mTORC2 in modulating other placental functions is largely unexplored. We used a gene array following the silencing of rictor to identify genes regulated by mTORC2 in primary human trophoblast (PHT) cells. Four hundred and nine genes were differentially expressed; 102 genes were down-regulated and 307 up-regulated. Pathway analyses demonstrated that inhibition of mTORC2 resulted in increased expression of genes encoding for pro-inflammatory IL-6, VEGF-A, leptin, and inflammatory signaling (SAPK/JNK). Furthermore, down-regulated genes were functionally enriched in genes involved in angiogenesis (Osteopontin) and multivitamin transport (SLC5A6). In addition, the protein expression of leptin, VEGFA, IL-6 was increased and negatively correlated to mTORC2 signaling in human placentas collected from pregnancies complicated by intrauterine growth restriction (IUGR). In contrast, the protein expression of Osteopontin and SLC5A6 was decreased and positively correlated to mTORC2 signaling in human IUGR placentas. In conclusion, mTORC2 signaling regulates trophoblast expression of genes involved in inflammation, micronutrient transport, and angiogenesis, representing novel links between mTOR signaling and multiple placental functions necessary for fetal growth and development.

## Introduction

The intrauterine environment impacts the lifelong health of the fetus (Barker et al., 1993; Gluckman and Hanson, 2004a,b; Gluckman et al., 2008). The placenta plays a critical role in regulating the intrauterine environment and orchestrating fetal growth and organ-specific development (Burton and Jauniaux, 2015; Burton et al., 2016). The placenta mediates the transport of nutrients and oxygen from the mother to the fetus, provides immune protection, and secretes hormones into the maternal and fetal circulations. Emerging evidence suggests that the placenta functions as a nutrient sensor which integrates maternal and fetal nutritional cues with information from intrinsic nutrient-sensing signaling pathways to regulate placental nutrient transport and fetal growth (Jansson and Powell, 2007; Burton and Jauniaux, 2015). Therefore, changes in the maternal compartment or in the intrauterine environment caused by various intrinsic and extrinsic stressors modulate placental function, including nutrient transport, blood flow, metabolism, and hormone secretion. These changes in placental function may adversely impact the developing fetus with potential health consequences across the lifespan (Sandovici et al., 2012). However, the molecular mechanisms regulating the function of the human placenta are poorly understood (Guttmacher et al., 2014).

The mechanistic target of rapamycin (mTOR) is an evolutionarily conserved signaling hub that belongs to the phosphatidylinositol kinase-related kinase (PIKK) family (Wullschleger et al., 2006). mTOR exists as two functionally and structurally different complexes, mTORC1 and mTORC2 (Wullschleger et al., 2006). One key difference between the two complexes is that the protein Raptor is associated with mTORC1 and the protein Rictor constitutes a part of mTORC2 (Kim et al., 2002; Sarbassov et al., 2004). Pharmacological and gene silencing studies have demonstrated that the activation of mTORC1 phosphorylates S6K1 and 4E-BP1, and promotes gene transcription and protein translation (Yang and Guan, 2007). mTORC2 is known to phosphorylate Akt, SGK1, and PKCα and regulates metabolism and cytoskeletal trafficking (Guertin et al., 2006). Placental mTOR signaling activity has been reported to be inhibited in human (Chen et al., 2015) and animal models of IUGR (Rosario et al., 2011; Kavitha et al., 2014). Moreover, activation of placental mTOR signaling is positively associated with fetal overgrowth in obese women (Jansson et al., 2013), and in a diet-induced mouse model of maternal obesity with increased fetal growth (Rosario et al., 2015b). Importantly, mTOR has emerged as a master regulator of placental function. Specifically, both mTORC1 and 2 are positive regulators of trophoblast amino acid and folate transport (Rosario et al., 2015a, 2016a,b) and O-linked N-acetylglucosamine (O-GlcNAc) transferase (OGT) protein expression (Kelly et al., 2020), whereas mTORC1 activation promotes placental mitochondrial biogenesis/respiration (Rosario et al., 2013). We also demonstrated that mTORC 2 signaling is a negative regulator of trophoblast serotonin synthesis (Kelly et al., 2020). However, mTORC2 regulation of other trophoblast functions remains largely unknown.

We recently reported that mTORC1 regulates > 700 genes in cultured primary human trophoblast (PHT) cells (Rosario et al., 2020). Specifically, inhibition of mTORC1 down-regulates trophoblast genes involved in ribosome subunits, protein synthesis, and molecular transport (Rosario et al., 2020). In contrast, mTORC2 regulation of the trophoblast transcriptome has not been previously explored. In the present study, we employed an unbiased discovery approach to find regulatory networks and novel regulators that can help us decipher mTORC2 regulation of gene expression in PHT cells.

## Materials and Methods

### Ethical Approval

The Institutional Review Board at the University of Texas Health Science Center, San Antonio, approved all the experimental protocols. For the gene expression profiling experiment, placentas of uncomplicated term pregnancies were collected with informed consent at the Labor and Delivery Unit at the University Hospital San Antonio. Selected clinical characteristics of the study subjects are provided in Table 1.

**TABLE 1:** Selected clinical data of the mothers and newborns from whom placental samples were obtained for the transcriptomics analysis.

	**Placenta 1**	**Placenta 2**	**Placenta 3**	**Placenta 4**
Maternal age (years)	26.0	27.0	25.0	28.0
BMI (kg/m^2^) *	24.5	23.5	22.5	23.0
Gestational age (weeks)	37.1	37.2	37.3	37.5
Birth weight (g)	2,500	2,645	2,589	2,679
Placental weight (g)	645	680	595	604
Fetal sex (M/F)	M	F	M	F
Mode of delivery (C/V)	C	C	C	C

F, female; M, male; C, cesarean section; V, vaginal delivery. *Maternal BMI (kg/m2) was calculated using maternal weight and height measurements taken during the first (10–12 weeks) trimesters.

### Isolation and Culture of Primary Human Trophoblast Cells

Placentas were collected immediately following delivery by cesarean section at term without labor. As described previously, primary PHT cells were isolated by trypsin digestion followed by discontinuous Percoll gradient separation (Rosario et al., 2016a). PHT cells were plated in 60 mm culture dishes (∼7.5 × 10^6^ cells/dish) and cultured in 5% CO2, 95% atmosphere air at 37°C for 90 h. Cell culture media (DMEM/Hams F-12) containing L-glutamine, penicillin, streptomycin, gentamycin, and 10% fetal bovine serum was changed daily.

### RNA Interference-Mediated Silencing in Primary Human Trophoblast Cells

RNA interference-mediated silencing of the target gene was performed in PHT cells as previously described (Rosario et al., 2013). Briefly, Dharmafect 2 transfection reagent (Thermo Fisher Scientific, Rockford, IL) and small interfering RNAs (siRNAs) (Sigma-Aldrich, St. Louis, MO) targeting *RICTOR* (100 nM: sense, 5’ CGAUCAUGGGCAGGUAUUA) were used (Supplementary Figure 1). First, we searched BLASTn to ensure that the designed *RICTOR* siRNA sequences would not target any other gene transcript. Then, control PHT cells were transfected with a non-coding scrambled sequence (100 nM; sense: 5’GAUCAUACGUGCGAUCAGATT). The *RICTOR* or non-coding scrambled sequence siRNAs, synthesized by Sigma-Aldrich, were used according to the manufacturer’s instructions (with the final concentration of 100 nM). According to the manufacturer’s instructions, after 18 h in culture, PHT cells were transfected with siRNAs using Dharmafect 2 transfection reagent. PHT cells were incubated in siRNA for 24 h and then removed, fresh medium was added to wells (Forbes et al., 2009). At 90 h in culture, *RICTOR* silencing efficiency was assessed at the protein (expression of rictor) and functional levels (phosphorylation of mTORC2 downstream target; AKT-Serine-473) using Western blot.

### Assessment of Biochemical Differentiation and Viability

The human chorion gonadotropin (hCG) is a marker of trophoblast cell differentiation and viability. Using the commercially available ELISA kit from Immuno Biological Labs, we quantified the secretion of hCG (kit designed to detects the beta subunit of hCG) in conditioned media collected at 18, 42, 66, and 90 h after plating PHT cells. Using Western blot, we also measured the protein expression of caspase-3 (a marker of apoptosis) in the cell lysates of scramble and Rictor silenced PHT cells.

### RNA Isolation From Primary Human Trophoblast Cells

According to the manufacturer’s instructions, RNA was isolated from cultured PHT cells at 90 h in culture using TRIzol Reagent (Invitrogen, Carlsbad, CA). RNA was resuspended in 100 μl DEPC-treated water. RNA quality was determined using an Agilent 2100 Bioanalyzer (Agilent Technologies, Inc., Santa Clara, CA), and RNA concentrations were confirmed by quantitation using a NanoDrop^TM^ 8000 spectrophotometer (Thermo Fisher Scientific, Wilmington, DE).

### Gene Expression Profiling in Primary Human Trophoblast Cells

Whole-genome expression profiling was performed using gene arrays (HumanHT-12 v4 Expression BeadChips, Illumina Inc., San Diego, CA). cRNA was synthesized and biotin-labeled according to the manufacturer’s instructions (cat. no. 1750, Ambion, Austin, TX). Total RNA was used for first and second-strand cDNA synthesis, followed by *in vitro* transcription to synthesize biotin-labeled cRNA. cRNA was quality checked and then hybridized to Human HT-12 v4 Expression BeadChips (Illumina Inc.). Individual cRNA samples were used to interrogate each BeadChip (Scramble siRNA, *n* = 4; *RICTOR* siRNA, *n* = 4). Gene expression was detected and cleaned using GenomeStudio software (Illumina Inc.) and filtered using a quality score (>0.95). Gene array data were all-median normalized and log_2_ transformed (GeneSifter), and differentially expressed genes were identified by *t*-test (*p* < 0.05).

### Pathway Analysis

Using GeneSifter, genes with significantly different expression levels were superimposed on Kyoto Encyclopedia of Genes and Genomes (KEGG) pathways (Kanehisa and Goto, 2000; Kanehisa et al., 2016, 2017). In addition, GeneSifter was used to compute Z-scores, which is described in detail in Supplementary Methods.

### Network Analysis

The networks were generated through the use of IPA (QIAGEN Inc.)^1^ (Kramer et al., 2014). Network analysis (Ingenuity Pathway Analysis (IPA), Ingenuity^®^ Systems, Redwood City, CA) was performed using differentially expressed genes (*p* < 0.05) from each pairwise comparison (RICTOR vs. Scramble). Networks were built using the IPA Knowledge Base, using expression profiles from this dataset and requiring direct connections between molecules based on experimental evidence (Kramer et al., 2014). Network significance was calculated in IPA using Fisher exact *t*-test (Ingenuity^®^ Systems). The *p*-value for a given network takes into account the number of eligible molecules (differentially expressed genes) in the selected reference set (defined by the Ingenuity Knowledge Base); the total number of molecules in the selected reference set known to be associated with that function; the total number of eligible molecules in the selected reference set; and the total number of molecules in the reference set (Ingenuity^®^ Systems). This analysis considered networks are containing > 25 differentially expressed genes and a *p*-value < 10^–20^ as significant.

### Placental Mechanistic Target of Rapamycin Complex 2 Signaling and Expression of Leptin, VEGF-A, IL-6, Osteopontin, and Sodium-Dependent Multivitamin Transporter (SLC5A6) in Intrauterine Growth Restriction

Placentas from pregnancies complicated by IUGR and women delivering appropriate-for-gestational age (AGA) infants were collected within 15 min of delivery as described (Chen et al., 2015). The study was approved by the University of Western Ontario Health Sciences Research Ethics Board. Pregnant women attending St. Joseph’s Health Care Centre, London, Ontario, Canada, were enrolled after informed consent was obtained. The decidua basalis and chorionic plate were removed, and villous tissue was dissected and rinsed in cold physiological saline. The villous tissue was transferred to cold buffer D (250 mM sucrose, 10 mM HEPES, pH 7.4) containing 1:100 dilution of protease and phosphatase inhibitors (Sigma–Aldrich, St. Louis, MO, United States) and homogenized on ice with a Polytron (Kinematica, Luzern, Switzerland). Placental homogenates were frozen in liquid nitrogen and stored at –80°C until further processing. The phosphorylation of key proteins in the mTORC2 (Chen et al., 2015) and leptin, VEGF-A, IL-6, osteopontin, and SLC5A6 expression in placental homogenates of IUGR and AGA groups were determined using Western blots as described previously (Rosario et al., 2016a).

### Statistics

The number of experiments (n) denotes the number of placentas studied. Data are represented as means ± S.E.M. All the array data from each sample were all-median normalized and log_2_ transformed. Box plots were tested to ensure that each group’s median was 0 and variance among groups was similar. Statistical analyses of array data were performed by *t*-test using GeneSifter software (Geospiza, Inc.) for pairwise comparisons. Statistical significance of differences between control and experimental groups in protein expression studies were assessed using Student’s *t*-test. A *P*-value < 0.05 was considered significant.

## Results

### Rictor Silencing in Primary Human Trophoblast Cells Inhibits mTORC2 Signaling

**FIGURE 1:**
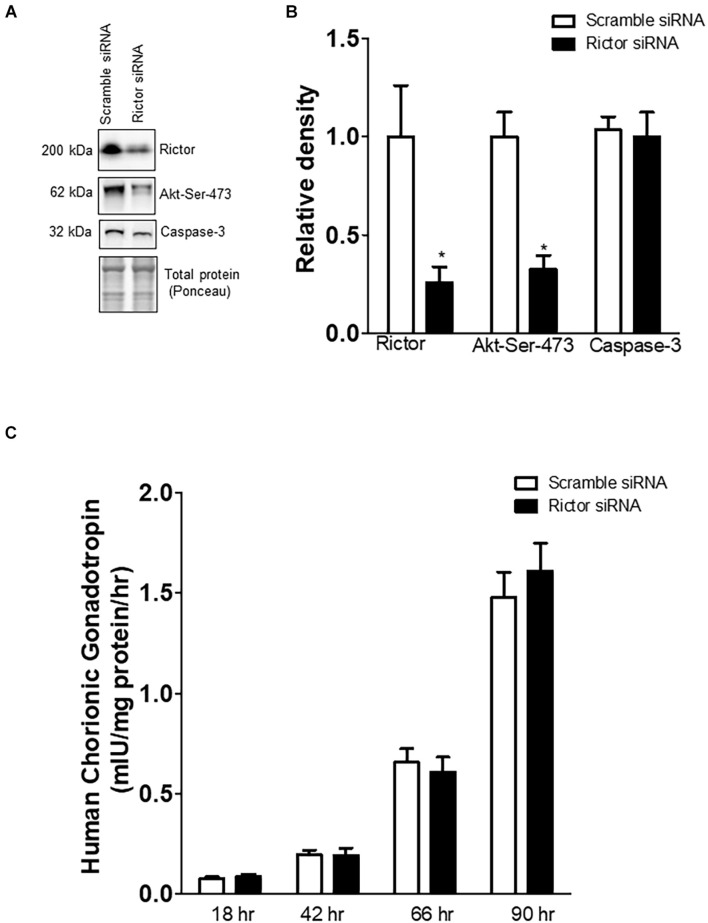
Effect of rictor silencing on rictor protein expression, mTORC2 signaling activity, and trophoblast differentiation and viability. **(A)** Representative western blots of rictor, phosphorylated Akt-Ser-473 and caspase-3 expression in cell lysates of scramble siRNA and rictor siRNA silenced PHT cells. Equal loading was performed. **(B)** Summary of the western blot data of rictor, Akt-Ser-473, and caspase-3 protein. **(C)** Secretion of human chorionic gonadotropin (hCG) from PHT cells transfected with scramble or rictor siRNA. Values are given as means + SEM. *P < 0.05 vs. scramble siRNA; unpaired Student’s *t*-test; *n* = 5/each group.

Rictor siRNA markedly decreased the protein expression of rictor (–75%, *p* = 0.001; *n* = 5/each group) and decreased the phosphorylation of Akt-Ser-473 (–68%, *p* = 0.003; *n* = 5/each group), a functional readout for mTORC2 signaling (Figure 1). However, total Akt protein expression was comparable between scramble and Rictor siRNA silenced PHT cells (Rosario et al., 2013). The hCG secretion profiles and caspase-3 protein expression were comparable between PHT cells transfected with scrambled or rictor siRNA (Figure 1, *n* = 5/each group). These findings indicate that mTORC2 inhibition did not affect differentiation/syncytialization and viability of cultured PHT cells and suggest that rictor silencing effects on the trophoblast transcriptome were not caused by unspecific effects on differentiation. We previously demonstrated that there is no cross talk between mTORC1 and mTORC2 signaling in PHT cells (Rosario et al., 2013).

### Analysis of Differentially Expressed Genes in Response to mTORC2 Inhibition Signaling in Primary Human Trophoblast Cells

**FIGURE 2:**
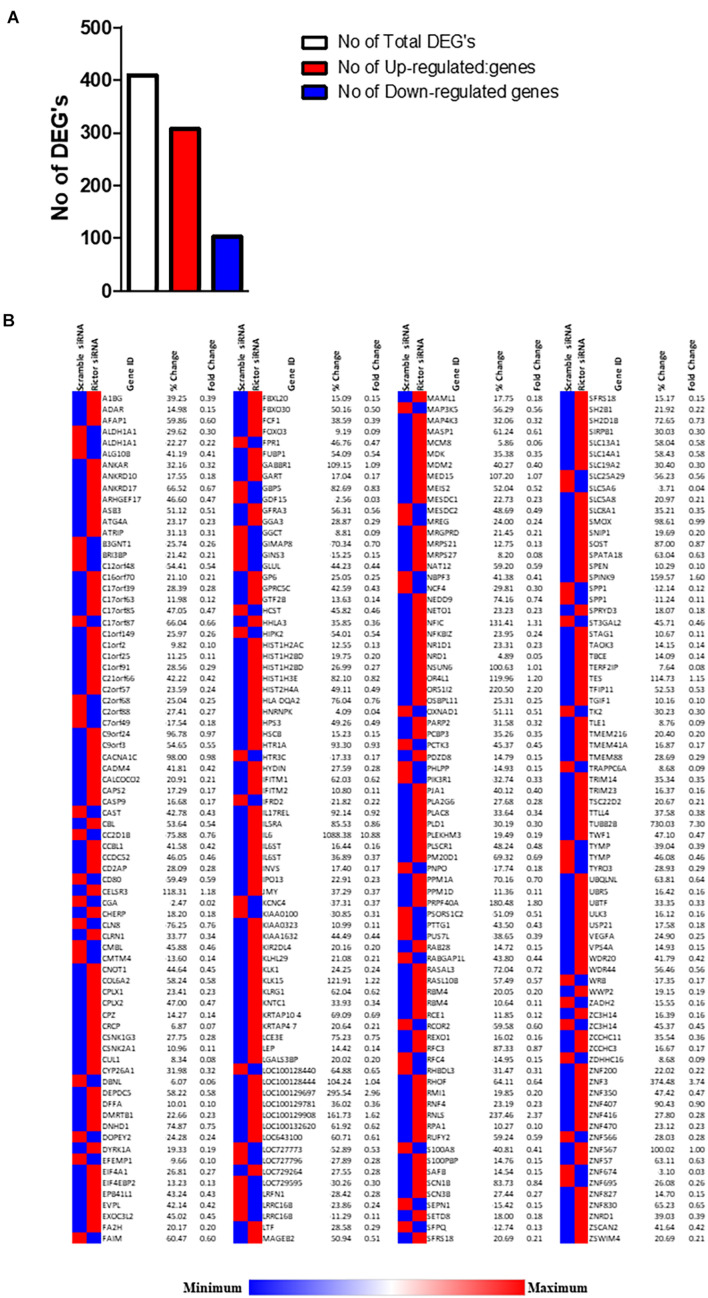
Differentially expressed genes in rictor siRNA vs. scramble siRNA silenced PHT cells. **(A)** The number of differentially expressed genes in rictor siRNA vs. scramble siRNA silenced PHT cells. The red represents up-regulated genes and blue represents down-regulated genes in rictor silenced PHT cells as compared to cells transfected with scramble siRNA. **(B)** Heatmap of expression data for the differentially expressed genes in rictor siRNA vs. scramble siRNA silenced PHT cells. Color index represents gene expression changes, the red represents up-regulated genes, and the blue represent down-regulated genes in rictor silenced PHT cells as compared to cells transfected with scramble siRNA.

We analyzed DEGs following *rictor* siRNA silencing (mTORC2 inhibition), all sequenced genes were compared between rictor and scramble siRNA silenced (Rictor vs. scramble). We identified a total of 409 DEGs between rictor and scramble siRNA treated PHT cells, comprising 307 up-regulated and 102 downregulated DEGs (Figure 2 and Supplementary Table 1). Differentially expressed genes are displayed in the heat map in Figure 2.

Interestingly, we found several ubiquitin-conjugating E2 transcripts, such as UBE2D3 and E3 ubiquitin-protein ligase (UBR5, UBE2D3, PJA2, RBCK1, RNF38, TRIM5, TRIM27, TRIM23, TRIM9, and TRIM28) were up-regulated following rictor silencing (Supplementary Table 1). Furthermore, mTORC2 inhibition increased the expression of ubiquitin-specific proteases such as USP21, USP2, USP9X, USP53, USP5, and USP49.

### Functional Analysis of Differentially Expressed Genes

Next, we identified the Gene Ontology (GO) terms enriched with differentially expressed genes in the biological process (BP). Separate GO enrichment analyses for up-regulated and down-regulated genes were performed. GO analysis revealed that up-regulated genes were mainly involved in biological processes, including response to stress, regulation of response to the stimulus, response to an external stimulus, regulation of biological quality, and cytokine-mediated signaling pathway (Table 2). In contrast, down-regulated genes primarily played a role in regulating signaling, regulation of molecular function, immune system process, immune response, and immune system regulation (Table 3).

**TABLE 2:** Gene Ontology biological process classification of up-regulated genes in rictor silenced PHT cells.

**N**	**High level GO category**	**Genes**
66	Response to stress	MAP4K3 ERCC8 DEPDC5 UBR5 VEGFA MASP1 SEM1 PARP2 RPA1 ANKRD17 CASP9 TAOK3 EVPL LEP RMI1 IFITM2 IFITM1 ZNF830 PARG TFIP11 IL6ST LTF TRIM14 MCM8 MDM2 IL6 CALCOCO2 TRPM2 SPATA18 RBM4 PLA2G6 KIR2DL4 NCBP3 IL5RA FBXL20 CBL MDK TRIM23 FOXO3 KLRG1 NFKBIZ PLAC8 JMY DYRK1A ADAR ATRIP HNRNPK TERF2IP PPM1D HTR1A SLC8A1 RNLS PLSCR1 TSC22D2 SH2D1B CRCP GP6 NR1D1 KMT5A CUL1 LGALS3BP CNOT1 RFC3 ULK3 SH2B1 HLA-DQA2
62	Regulation of response to stimulus	PPM1A DEPDC5 CBL VEGFA MASP1 ZSWIM4 IL6ST TAOK3 CSNK1G3 TMEM88 LEP TLE1 PARG IL6 LTF INVS CNOT1 ANKRD17 MDM2 ULK3 NETO1 SOST RASGRP4 KIR2DL4 MAP4K3 ARHGEF17 MDK FOXO3 NR1D1 PARP2 TUT4 TRPM2 NFKBIZ TWF1 DYRK1A NPR2 HNRNPK TERF2IP SH2B1 PLA2G6 AFAP1 CHERP CSNK2A1 UBR5 RASAL3 CASP9 TUBB2B ADAR KMT5A PLSCR1 CD2AP TFIP11 SH2D1B CYP26A1 RPA1 RHOF JMY MAML1 MEAF6 IFITM1 WWP2 HLA-DQA2
53	Regulation of biological quality	LTF ZC3H14 SCN1B FOXO3 RPA1 RHOF CPLX2 TWF1 JMY SCN3B TERF2IP KLK1 CPLX1 LEP PLSCR1 TFIP11 SLC8A1 CHERP CYP26A1 VEGFA GGA3 MDM2 IL6 CACNA1C PDZD8 RNLS WWP2 NR1D1 TRPM2 PLAC8 EIF4EBP2 SAFB PM20D1 NETO1 HTR1A RMI1 PLA2G6 ZNF830 GABBR1 RASL10B GP6 TMEM88 FA2H MCM8 OSBPL11 ADAR CLRN1 PRPF40A NRDC CUL1 RFC3 NPR2 SH2B1
41	Response to external stimulus	OPN3 DEPDC5 VEGFA ZSWIM4 LEP IFITM2 IFITM1 IL6ST IL6 LTF ANKRD17 ADAR RASGRP4 PLA2G6 CELSR3 ERCC8 NCBP3 CYP26A1 SCN1B CBL FOXO3 CASP9 MEIS2 TUT4 MDM2 TRPM2 PLAC8 GFRA3 PPM1D KYAT1 RMI1 SLC8A1 PLSCR1 CRCP SOST NR1D1 CALCOCO2 TUBB2B NFKBIZ RBM4 PLD1
13	Cytokine-mediated signaling pathway	ADAR IL5RA IFITM1 CUL1 CBL HLA-DQA2 LEP IL6ST IL17REL FOXO3 IFITM2 IL6

*The ShinyGO application (version 0.66) (Ge et al., 2020) was used for exploring enrichment in Gene Ontology (GO) categories for biological processes using the 150 top genes obtained from the gene-based association analyses. Multiple testing correction was applied using the Benjamini–Hochberg method implemented in the application. We considered significant those processes with false discovery rate (FDR) p-value < 0.05. Only categories with a minimum of 10 overlapping genes were selected. N: is the total number of genes the total numbers of genes included on the array and giving a signal in the primary trophoblast cultures. The number of experiments (n) denotes the number of placentas studied, n = 4 placenta/each group, rictor siRNA and scramble siRNA.*

**TABLE 3:** Gene Ontology biological process classification of down-regulated genes in rictor silenced (mTORC2 inhibited) PHT cells.

**N**	**High level GO category**	**Genes**
22	Regulation of signaling	PHLPP1 HCST GDF15 NEK10 MAP3K5 SFPQ SPP1 CGA S100A8 SCIMP CRLF2 TYMP TYRO3 EFEMP1 KCNC4 GLUL PIK3R1 HIPK2 SELENON CD80 DBNL FPR1
21	Regulation of molecular function	PIK3R1 RABGAP1L CAST NEK10 MAP3K5 GDF15 CGA S100A8 RFC4 TRAPPC6A TYMP HIPK2 NCF4 EFEMP1 SPP1
14	Immune system process	CD80 FPR1 SFPQ S100A8 PHLPP1 TYRO3 HCST DBNL PIK3R1 GBP5 MAP3K5 CRLF2 HIPK2 NCF4
11	Immune response	CD80 FPR1 SFPQ TYRO3 HCST DBNL S100A8 GBP5 MAP3K5 NCF4 PIK3R1
10	Regulation of immune system process	CD80 FPR1 SFPQ PHLPP1 TYRO3 HCST PIK3R1 GBP5 CRLF2 S100A8

*The ShinyGO application (version 0.66) (Ge et al., 2020) was used for exploring enrichment in Gene Ontology (GO) categories for biological processes using the 150 top genes obtained from the gene-based association analyses. Multiple testing correction was applied using the Benjamini–Hochberg method implemented in the application. We considered significant those processes with false discovery rate (FDR) p-value < 0.05. Only categories with a minimum of 10 overlapping genes were selected. N: is the total number of genes the total numbers of genes included on the array and giving a signal in the primary trophoblast cultures. The number of experiments (n) denotes the number of placentas studied, n = 4 placenta/each group, rictor siRNA and scramble siRNA.*

### Ingenuity Pathway Analysis

**FIGURE 3:**
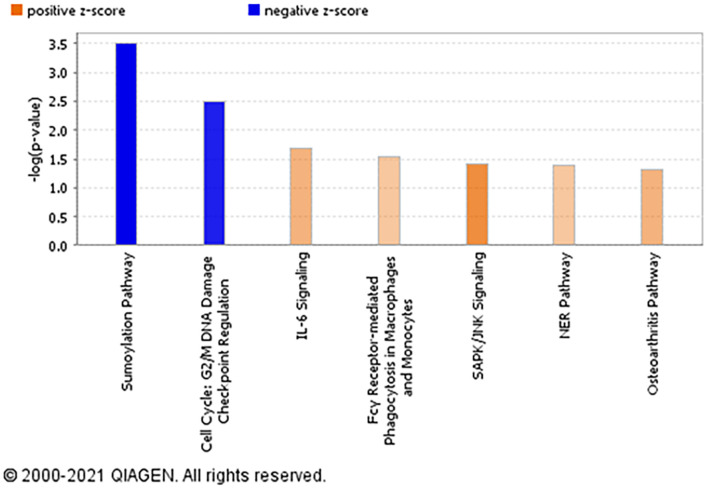
Overall functional analysis. Analysis of the top molecular and cellular functions determined by IPA for the rictor siRNA silenced (mTORC2 inhibited) PHT cells. Ingenuity Pathway Analyses (IPA) of array data identified canonical pathways significantly affected by rictor silencing in PHT cells. Color index represents pathway expression changes, the orange represents up-regulated pathways, and the blue represent down-regulated pathways in rictor silenced PHT cells as compared to cells transfected with scramble siRNA.

The top significantly enriched canonical signaling pathways up-regulated in rictor-silenced PHT cells were IL-6 (Interleukin-6), SAPK/JNK (Stress-activated protein kinase/c-Jun NH(2)-terminal kinase), and NER (Nucleotide excision repair) signaling (Figure 3). Conversely, we found that sumoylation and cell cycle G2/M DNA damage checkpoint regulation pathways were down-regulated in mTORC2 inhibited PHT cells (Figure 3).

**FIGURE 4:**
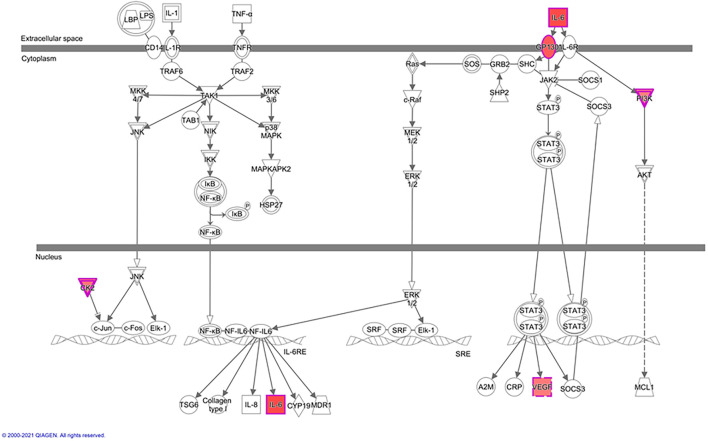
Rictor silencing up regulated components of IL-6 signaling in PHT cells. Graphical representation of PHT cell IL-6 signaling networks identified by Ingenuity Pathway Analysis (IPA) following rictor silencing. Molecular relationships between genes up-regulated (red) or no change (white) in expression after rictor silencing are shown; CSNK2A1, Casein kinase 2 alpha 1; IL6, Interleukin 6; IL6ST, Interleukin 6 signal transducer; PIK3R1, Phosphoinositide-3-kinase regulatory subunit 1; VEGFA, Vascular endothelial growth factor A.

In greater detail, we used IPA-canonical pathway analysis to study mTORC2 regulation of genes encoding for IL-6 signaling proteins. We found that five genes were identified as significantly activated in rictor silenced PHT cells. Specifically, IL6, IL6ST, CSNK2A1, VEGFA, and PIK3R1 were up-regulated (Figure 4), suggesting that inhibition of mTORC2 results in a broad and coordinated up-regulation of genes encoding proteins involved in IL-6 signaling.

### Kyoto Encyclopedia of Genes and Genomes Pathway Analysis

KEGG pathway analysis comparing rictor/mTORC2 siRNA with scramble siRNA revealed 53 down-regulated and 63 up-regulated pathways. Down-regulated trophoblast pathways in response to mTORC2 inhibition included alanine, aspartate and glutamate metabolism, carbohydrate digestion and absorption, DNA replication, drug metabolism, leukocyte trans-endothelial migration, metabolic pathways, N-Glycan biosynthesis, neurotrophin signaling pathway, nitrogen metabolism, nucleotide excision repair, pyrimidine metabolism, toll-like receptor signaling pathway, type I diabetes mellitus, type II diabetes mellitus, and vitamin B6 metabolism (Table 4). Jak-STAT signaling pathway, mismatch repair, nucleotide excision repair, p53 signaling pathway, regulation of autophagy, ubiquitin-mediated proteolysis, and VEGF signaling pathways are examples of pathways up-regulated in response to mTORC2 inhibition (Table 5).

**TABLE 4:** Down-regulated KEGG pathways in PHT cells in response to rictor siRNA silencing.

**KEGG Pathways Down**	**Diff regulated genes**	**Up**	**Down**	**Gene set**	**Z-score**
Alanine, aspartate and glutamate metabolism	1	0	1	31	2.72
Aldosterone-regulated sodium reabsorption	1	0	1	41	2.27
Allograft rejection	2	1	1	33	2.62
Autoimmune thyroid disease	3	1	2	48	4.5
Bladder cancer	3	2	1	41	2.27
Carbohydrate digestion and absorption	1	0	1	42	2.24
DNA replication	3	2	1	35	2.52
Drug metabolism—other enzymes	2	0	2	41	4.93
Glycosaminoglycan biosynthesis—keratan sulfate	2	0	2	15	8.53
Glycosphingolipid biosynthesis—ganglio series	1	0	1	15	4.15
Glycosphingolipid biosynthesis—globo series	1	0	1	14	4.31
Glycosphingolipid biosynthesis—lacto and neolacto series	1	0	1	25	3.1
Graft-vs.-host disease	3	2	1	35	2.52
Intestinal immune network for IgA production	3	2	1	44	2.17
Leukocyte transendothelial migration	2	0	2	115	2.55
Metabolic pathways	14	5	9	1,080	2.99
Mucin type O-Glycan biosynthesis	1	0	1	30	2.78
N-Glycan biosynthesis	1	0	1	49	2.01
Natural killer cell mediated cytotoxicity	4	2	2	133	2.28
Neurotrophin signaling pathway	4	2	2	127	2.37
Nitrogen metabolism	1	0	1	23	3.25
Nucleotide excision repair	4	3	1	43	2.2
Osteoclast differentiation	3	1	2	127	2.37
Pyrimidine metabolism	3	1	2	95	2.92
Toll-like receptor signaling pathway	4	1	3	102	4.47
Type I diabetes mellitus	2	1	1	38	2.39
Type II diabetes mellitus	2	1	1	47	2.07
Vitamin B6 metabolism	1	0	1	5	7.45

*Table lists KEGG pathways that were significantly downregulated in PHT cells with rictor silencing as compared to control cells; “List” denotes the total number of differentially expressed genes on the array. “Up” denotes the number of up-regulated genes; “Down” denotes the number of down-regulated genes; “Gene set” denotes the total number of genes in this pathway that are included on the array and give a signal with PHT cell RNA; and “Z-score” denotes the z-score for the list pathway. The significance of identified KEGG pathways was determined by Z-score. Pathways were considered significantly different between scramble and Rictor siRNA groups if the Z-score for that pathway was > 2.00. The number of experiments (n) denotes the number of placentas studied, n = 4 placenta/each group, rictor siRNA and scramble siRNA.*

**TABLE 5:** Up-regulated KEGG pathways in PHT cells in response to rictor silencing.

**KEGG pathways up**	**List**	**Up**	**Down**	**Gene set**	**z-score (Up)**
Circadian rhythm—mammal	2	2	0	22	3.36
Ether lipid metabolism	2	2	0	33	2.53
Jak-STAT signaling pathway	6	5	1	154	2.31
Mismatch repair	3	2	1	22	3.36
Nucleotide excision repair	4	3	1	43	3.44
p53 signaling pathway	3	3	0	68	2.41
Pancreatic cancer	4	3	1	70	2.34
Regulation of autophagy	2	2	0	33	2.53
Systemic lupus erythematosus	6	5	1	130	2.75
Ubiquitin mediated proteolysis	6	6	0	135	3.44
VEGF signaling pathway	4	3	1	73	2.26

*Table lists KEGG pathways that were significantly up regulated in PHT cells with rictor silencing as compared to control cells; “List” denotes the total number of differentially expressed genes on the array. “Up” denotes the number of up-regulated genes; “Down” denotes the number of down-regulated genes; “Gene set” denotes the total number of genes in this pathway that are included on the array and give a signal with PHT cell RNA; and “Z-score” denotes the z-score for the list pathway. The significance of identified KEGG pathways was determined by Z-score. Pathways were considered significantly different between scramble and Rictor siRNA groups if the Z-score for that pathway was > 2.00. The number of experiments (n) denotes the number of placentas studied, n = 4 placenta/each group, rictor siRNA and scramble siRNA.*

### Network Analysis

Network analysis of RNA expression from PHT cells with mTORC2 inhibition (rictor silencing) compared with control cells (scramble) revealed three networks (Supplementary Figures 4–6). The top annotated functions of the genes composing the networks included: RNA post-transcriptional modification, post-translational modification, tissue development, cell morphology, cellular function and maintenance, cancer, lipid metabolism, molecular transport, small molecule biochemistry, DNA replication, recombination, and repair, cell cycle, gene expression, cellular movement, inflammatory response, endocrine system disorders. Evaluation of these networks showed a coordinated response to Rictor silencing with the majority of genes up-regulated.

### Comparison Between mTORC1 and mTORC2 Transcriptome

Despite how clear it is that mTOR has diverse functions in placenta, the comparative roles of both mTOR complexes in PHT cells gene expression regulation specifically are poorly understood. Next, we compared it with the mTORC1 transcriptome (Rosario et al., 2020). The common differential expressed genes between mTORC1 and mTORC2 inhibition are listed in Table 6. GO analysis of common DEG’s identified the categories molecular function regulator (DEPDC5 SCN1B ARHGEF17 RABGAP1L SCN3B IL6 SPINK9 TYMP RASAL3 SPP1 PTTG1 ALDH1A1), transporter activity (SLC13A1 KCNC4 SLC5A6 SCN1B SCN3B SLC5A8), and transmembrane transporter activity (SLC13A1 KCNC4 SLC5A6 SCN1B SCN3B SLC5A8). In addition, sodium-dependent multivitamin transporter (SLC5A6) and osteopontin transcripts regulated transport activity and molecular function and were down-regulated in response to both mTORC1 and mTORC2 inhibition. It is interesting to observe that the two mTOR complexes have distinct and overlapping roles in transcription regulation.

**TABLE 6:** List of common DEG’s between mTORC1 and mTORC2 inhibited PHT cells.

**Gene ID**	**Gene name**	**Expression**	
		**mTORC1 inhibition**	**mTORC2 inhibition**
ALDH1A1	Aldehyde dehydrogenase 1 family member A1	Down	Down
ARHGEF17	Rho guanine nucleotide exchange factor 17	Up	Up
BRI3BP	BRI3-binding protein	Down	Down
C16ORF70	Chromosome 16 open reading frame 70	Up	Up
C9ORF24	Chromosome 9 open reading frame 24	Up	Up
CADM4	Cell adhesion molecule 4	Up	Up
CMBL	Carboxymethylenebutenolidase	Down	Down
CMTM4	CKLF like MARVEL transmembrane domain containing 4	Down	Down
CPLX2	Complexin 2	Up	Up
CPZ	Carboxypeptidase Z	Up	Up
DEPDC5	DEP domain containing 5, GATOR1 subcomplex subunit	Up	Up
DNHD1	Dynein heavy chain domain 1	Up	Up
EXOC3L2	Exocyst complex component 3-like 2	Up	Up
FA2H	Fatty acid 2-hydroxylase	Up	Up
FPR1	Formyl peptide receptor 1	Down	Down
GBP5	Guanylate-binding protein 5	Up	Up
GGCT	Gamma-glutamylcyclotransferase	Down	Down
GLUL	Glutamate-Ammonia Ligase	Down	Down
HLA-DQA2	Major histocompatibility complex, class II, DQ alpha 2	Up	Up
IL17REL	Interleukin 17 receptor E like	Up	Up
IL6	Interleukin 6	Up	Up
KCNC4	Potassium voltage-gated channel subfamily C member 4	Down	Down
KLK1	Kallikrein 1	Up	Up
KLK15	Kallikrein related peptidase 15	Up	Up
LGALS3BP	Galectin-3-binding protein	Up	Up
LRRC16B	Leucine rich repeat containing 1	Down	Down
MASP1	Mannan binding lectin serine peptidase 1	Up	Up
MCM8	Minichromosomal maintenance 8 homologous recombination repair factor	Up	Up
MDM2	Murine double minute 2	Up	Up
MREG	Melanoregulin	Down	Down
NETO1	Neuropilin and tolloid like 1	Up	Up
NFKBIZ	NFKB inhibitor zeta	Up	Up
OR4L1	Olfactory receptor family 4 subfamily L member 1	Up	Up
OXNAD1	Oxidoreductase NAD binding domain containing 1	Down	Down
PJA1	Praja ring finger ubiquitin ligase 1	Up	Up
PPM1A	Protein phosphatase 1A	Down	Up
PTTG1	PTTG1 Regulator of sister chromatid separation, securin	Down	Down
RABGAP1L	RAB GTPase activating protein 1 like	Down	Down
RASAL3	RAS protein activator like 3	Up	Up
RASL10B	RAS like family 10 member B	Up	Up
RCE1	Ras converting enzyme 1	Up	Up
SCN1B	Sodium voltage-gated channel beta subunit 1	Up	Up
SCN3B	Sodium voltage-gated channel beta subunit 3	Up	Up
SH2B1	SH2B adaptor protein 1	Up	Up
SLC13A1	Solute carrier family 13 (sodium/sulfate symporters)	Up	Up
SLC5A6	Solute carrier family 5 member 6	Down	Down
SLC5A8	Sodium-coupled monocarboxylate transporter 1	Up	Up
SMOX	Spermine Oxidase	Up	Up
SPINK9	Serine peptidase inhibitor kazal type 9	Up	Up
SPP1	Osteopontin	Down	Down
TYMP	Thymidine phosphorylase	Down	Down
UBQLNL	Ubiquitin like	Up	Up
ZC3H14	Zinc finger CCCH-type containing 14	Down	Down
ZNRD1	Zinc ribbon gene	Down	Up
SEPN1	Selenoprotein N, 1	Down	Down
PCTK3	PCTAIRE protein kinase 3	Down	Down
DOPEY2	Dopey family member 2	Down	Down
WRB	Tryptophan rich basic protein	Down	Down
MESD	Mesoderm development candidate 2	Down	Down

### Placental mTORC2 Signaling Is Associated With the Protein Expression of Leptin, VEGF-A, IL-6, Osteopontin, and Sodium-Dependent Multivitamin Transporter (SMVT, SLC5A6) Protein in Human Pregnancy

To explore the clinical relevance of our findings, we examined the relationship between placental mTORC2 signaling and protein expression of leptin, VEGF-A, IL-6, Osteopontin, and sodium-dependent multivitamin transporter (SLC5A6) in placentas collected from AGA and IUGR pregnancies. Selected clinical data for the AGA and IUGR groups are provided in Table 7. There was no significant difference in maternal age, body mass index (BMI), or gestational age between the control and the IUGR groups. However, birth weight was 28% lower (*P* < 0.01), and placental weight was reduced by 36% (*P* < 0.001) in the IUGR group compared with AGAs.

**TABLE 7:** Selected clinical data.

	**AGA (*n* = 19)**	**IUGR (*n* = 25)**
Maternal age (years)	25.9 ± 1.29	28.7 ± 1.23
BMI (kg/m^2^) *	28.3 ± 2.6	26.8 ± 2.0
Gestational age (weeks)	33.9 ± 0.95	35.7 ± 0.61
Birth weight (g)	2,493 ± 236	1,804 ± 110^†^
Birth weight percentile^‡^	55.9 ± 4.6	2.4 ± 0.3^§^
Placental weight (g)	566 ± 42.0	394 ± 18.4^——^
Fetal sex (M/F)	7/12	8/17
Mode of delivery (C/V)	6/13	15/10

Data are presented as means ± SEM. AGA, appropriate grown for gestational age; IUGR, intrauterine growth restriction; F, female; M, male; C, cesarean section; V, vaginal delivery. *Data from n = 10 AGA and 18 IUGR; ^‡^by corresponding gestational age; ^†^P < 0.05; ^∥^ P < 0.01; ^§^ P < 0.0001.

*Maternal BMI (kg/m^2^) was calculated using maternal weight and height measurements taken during the first (10–12 weeks) trimesters.*

**FIGURE 5:**
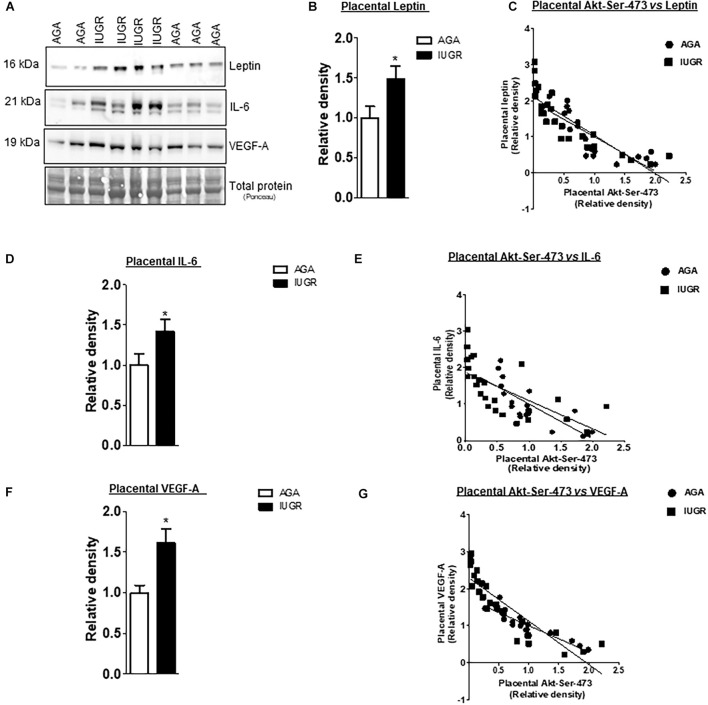
Correlation between placental mTORC2 functional readouts and the protein expression of leptin, VEGF-A and IL-6. **(A)** Representative western blots of leptin, IL-6 and VEGF-A expression in homogenates of AGA and IUGR placentas. Equal loading was performed. **(B,D,F)** Relative expression of leptin, VEGF-A and IL-6 in homogenates of AGA and IUGR placentas. *P < 0.05 vs. AGA, unpaired Student’s *t*-test. **(C,E,G)** Correlation between placental mTORC2 functional readouts AKT^Ser– 473^ and leptin, VEGF-A and IL-6 expression. r = Pearson correlation coefficient, n = AGA, 19; IUGR, 25. Pearson correlation analysis was used to examine the relation among the investigated factors (GraphPad Prism version 5). Leptin, AGA, *r* = 0.6403, *p* = 0.0001; IUGR *r* = 0.7121, *p* = 0.0001. VEGF-A, AGA, *r* = 0.8569, *p* = 0.0001; IUGR *r* = 0.7706, *p* = 0.0001. IL-6, AGA, *r* = 0.6560, *p* = 0.0001; IUGR *r* = 0.4379, *p* = 0.0001.

**FIGURE 6:**
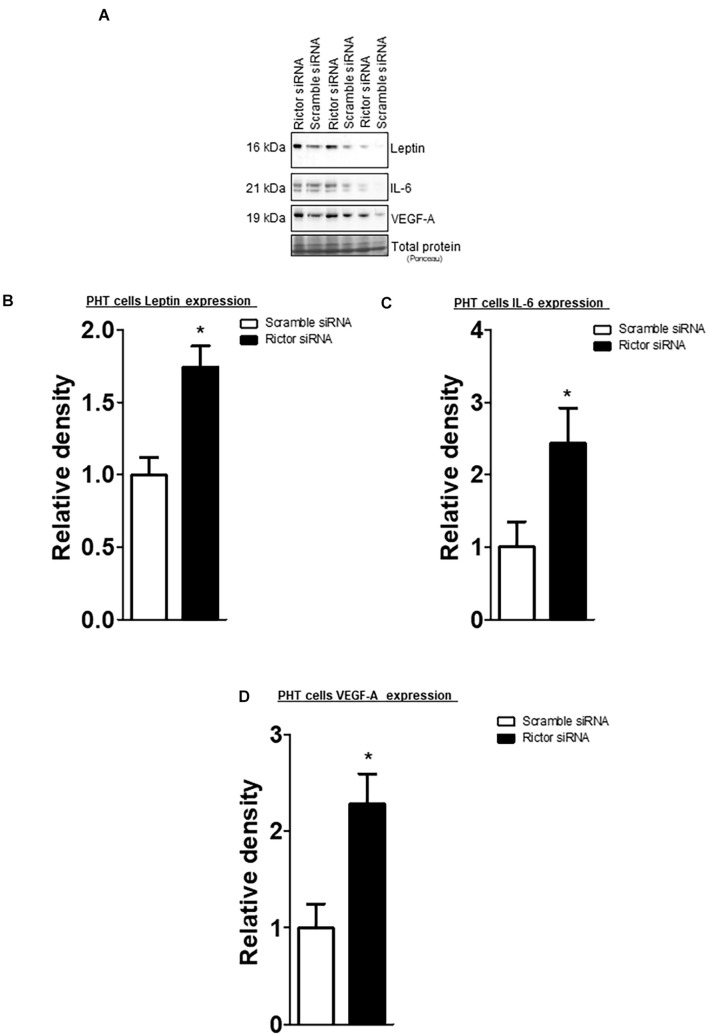
Effect of rictor silencing on leptin, IL-6 and VEGF-A protein expression in PHT cells. **(A)** Representative western blots of leptin, IL-6 and VEGF-A expression in cell lysates of scramble siRNA and rictor siRNA silenced PHT cells. Equal loading was performed. **(B–D)** Summary of the western blot data leptin, IL-6 and VEGF-A protein. Values are given as means + SEM. *P < 0.05 vs. scramble siRNA; unpaired Student’s *t*-test; *n* = 5/each group.

The leptin, VEGF-A, and IL-6 protein expression were significantly increased in IUGR placentas (Figure 5). However, osteopontin and sodium-dependent multivitamin transporter protein expression were significantly decreased in IUGR placentas (Figure 6). We previously reported phosphorylated Akt (Ser-473), a placental mTORC2 signaling functional readout, was reduced considerably in IUGR placentas compared to AGA (Chen et al., 2015). The protein expression of mTORC2 signaling was negatively correlated with leptin, VEGF-A, IL-6 in AGA, and IUGR placentas (Figure 5). In addition, we further validated the protein expression of leptin, VEGF-A, IL-6 in PHT cells silenced with scrmble and rictor siRNA by Western blotting as described in Supplementary Methods. As shown in Figure 6, silencing of rictor increased the protein expression of leptin (*p* = 0.003), VEGF-A (*p* = 0.01), IL-6 (*p* = 0.04) in PHT cells as compared to control (Scramble siRNA). This findings validate the transcriptome findings and also reinforce the translation of those findings with those from AGA and IUGR human placenta.

Additionally, the protein expression of mTORC2 signaling readout was positively associated with osteopontin and SLC5A6 in AGA and IUGR placentas (Figure 6).

## Discussion

Using gene silencing approaches, we report mTORC2 regulation of the trophoblast transcriptome in cultured primary human trophoblast cells, an experimental model system considered to be highly relevant to human pregnancy. mTORC2 inhibition predominantly resulted in up-regulation of genes (75% of the differentially expressed genes), and these genes encode primarily for pro-inflammatory (IL-6) and inflammatory signaling (SAPK/JNK). In contrast, genes involved in multivitamin transport were enriched among down-regulated genes following rictor silencing. Furthermore, placental gene expression of leptin, VEGF-A, and IL-6 were increased, whereas osteopontin and sodium-dependent multivitamin transporter were decreased following mTORC2 inhibition. To further demonstrate the clinical relevance of these findings, we show that mTORC2 signaling activity was negatively correlated to protein expression of leptin, VEGF-A, and IL-6 in placentas from pregnancies complicated by IUGR. In contrast, mTORC2 signaling activity was positively associated with osteopontin and sodium-dependent multivitamin transporter protein expression in placentas from pregnancies complicated by IUGR. This suggests that our findings in genetically manipulated cultured trophoblast cells have relevance to clinically important pregnancy complications.

Protein ubiquitination, which functions as a signal for trafficking proteins to the proteasome for subsequent degradation, appears to activate PHT cells following mTORC2 inhibition. Recent studies suggest that ubiquitin-specific proteases act as novel mTORC1 and −2 binding partners that negatively regulate mTOR activity (Agrawal et al., 2012). In addition, it has been previously shown that protein ubiquitination is altered in IUGR, specifically as a cause of increased oxidative stress, with accelerated degradation of p53 and Mcl-1 proteins, possibly contributing to placental insufficiency in IUGR (Rolfo et al., 2012).

We found increased LEP (leptin) gene expression among the most highly up-regulated genes by rictor siRNA silencing. IUGR is associated with increased placental leptin mRNA and leptin protein expression (Tzschoppe et al., 2011). Previous studies demonstrated that hypoxia is a potent stimulatory factor for placental leptin mRNA up−regulation (Grosfeld et al., 2002), mediated through a transcriptional mechanism likely to involve the HIF−1−dependent mechanism (Grosfeld et al., 2001). Leptin regulates placental growth, angiogenesis, and immune tolerance. The gene expression of *VEGF-A* (Vascular Endothelial Growth Factor A), which is known to play a role in vasculogenesis and angiogenesis, was up-regulated in response to mTORC2 inhibition. Previous studies demonstrated an elevated placental VEGF-A expression in IUGR pregnancy (Szentpeteri et al., 2013), which could be a secondary response to persistent hypoxia (Szentpeteri et al., 2013). Increased VEGF-A expression is associated with elevated placental syncytial knots (Azliana et al., 2017), a defining feature of maternal vascular mal-perfusion (Parks, 2018).

Moreover, global overexpression of VEGF-A resulted in severe abnormalities in heart development and embryonic lethality in mice (Miquerol et al., 2000). We demonstrated that protein expression of VEGF-A was higher in human IUGR placentas. It is possible that elevated VEGF-A expression in the IUGR placenta may decrease vascularization in placental villi and lead to reduced blood flow. Abnormal vascular development in the placental villi ultimately causes inadequate oxygen and nutrient transfer between mother and fetus. mTORC2 inactivation failed to mediate VEGF stimulated angiogenesis in endothelial cells (Farhan et al., 2015). Thus, mTORC2 signaling inhibition in IUGR placentas (Chen et al., 2015; Rosario et al., 2016a) is associated with elevated VEGF-A and leptin expression, which could be potentially responsible for the impaired angiogenesis in IUGR placentas. Collectively, these data implicate trophoblast mTORC2 in the regulation of placental angiogenesis.

We observed a significant increase in the transcript levels of genes encoding IL-6 signaling pathways in response to mTORC2 inhibition in PHT cells, which confirms previous findings demonstrating that loss of *rictor* in monocytes/macrophages activated IL-6 signaling (Babaev et al., 2018). Placental IL-6 mRNA and corresponding peptide levels were reported to be increased in IUGR pregnancy (Street et al., 2006). Additionally, IL-6 transcript increased in pregnancy complications associated with placental infection (Fedorka et al., 2020). Recent studies demonstrate that IL-6 activation in the placenta is necessary to relay inflammatory signals to the fetal brain and impact behaviors and neuropathologies relevant to neurodevelopmental disease (Wu et al., 2017). However, the lack of IL-6 signaling in trophoblasts effectively blocks maternal immune activation-induced inflammatory responses in the placenta and the fetal brain (Wu et al., 2017; Aguilar-Valles et al., 2020). Moreover, placental AKT/mTOR signaling is a possible mechanistic link to impaired neural circuit development and neurocognitive function (Howell and Law, 2020). We observed a negative correlation between placental mTORC2 signaling and IL-6 expression in placentas of IUGR pregnancies. Furthermore, we demonstrated that protein expression of IL-6 was higher in human IUGR placentas. We have previously shown that placental mTORC2 signaling is reduced in pregnancies complicated by IUGR (Chen et al., 2015) and speculate that the placenta’s aberrant mTORC2/IL6 signaling pathway may impair the fetal brain development in IUGR infants.

Osteopontin (SPP1), which belongs to the small integrin-binding ligand N-linked glycoprotein (SIBLING) family of extracellular matrix proteins and cytokines, has been reported to play multiple functions including cell proliferation, cell invasion, cell adhesion (Wu and Wang, 2016). It plays an essential role in embryo development in the mouse (Weintraub et al., 2004). Furthermore, osteopontin promotes invasion (Ke et al., 2020) and angiogenesis in the trophoblast and other cells (Li et al., 2021). Phosphatidylinositol 3-kinase (PI3K) is a heterodimer containing a regulatory subunit (p85) and a catalytic subunit (p110). The p85 regulatory subunit is essential for the p110 catalytic subunit’s stability and mediates the binding, activation, and localization of the PI3K enzyme (Luo et al., 2005). PI3K regulates growth in relation to nutrient supply (Engelman et al., 2006). In mice, inactivating p110α kinase causes growth-restriction at term (Foukas et al., 2006). Lopez-Tello et al. (2019) demonstrated that silencing of trophoblast p110α resulted in abnormal placental and fetal development. Furthermore, p110α kinase deficiency impairs the placenta from transporting nutrients to match fetal demands for growth. We also compared our current study pathway analysis with the existing transcriptome of trophoblast silenced with PI3K signaling (Lopez-Tello et al., 2019). Interestingly, pathways regulating immune function were down-regulated in both mTORC2 (current study) and PI3K signaling inhibited trophoblast cells (Lopez-Tello et al., 2019). These data suggest that mTORC2 and PI3K signaling may cause an immune imbalance in the placenta and result in fetal growth restriction (Wang et al., 2020; McColl and Piquette-Miller, 2021). We observed a decreased gene expression of the Osteopontin and PIK3R1 in *rictor* silenced cells. We have shown that mTORC2 signaling (Chen et al., 2015) and osteopontin (current study) expression is reduced in human IUGR placentas. We speculate that osteopontin and PI3K inhibition, mediated by reduced mTORC2 activity, may contribute to placental insufficiency and reduced fetal growth in IUGR.

Biotin is essential for normal fetal development and is provided to the fetus by transport across the placenta from the mother. Biotin transport in the human placenta is mediated by Na^+^-dependent multivitamin transporter (encoded by SLC5A6 gene) (Noam et al., 2020). Fetuses of biotin deficient mouse dams demonstrate fetal growth restriction (Levin et al., 1985). In the present study, we found a positive correlation between mTORC2 signaling and the protein expression of SLC5A6 in the IUGR placenta. We have also recently demonstrated that placental mTOR signaling regulates trophoblast folate transporter expression at the post-translational level by modulating the plasma membrane trafficking of specific transporter isoforms (Rosario et al., 2016b). Together with the demonstration in the current study that mTORC2 regulates specific trophoblast nutrient transporters at the transcriptional level, these findings suggest that trophoblast mTOR is a master regulator of a range of placental nutrient transporters mediated by distinct molecular mechanisms.

One potential limitation of using a single Rictor siRNA to inhibit the mTORC2 signaling could lead to off-target consequences as described elsewhere (Gagnon and Corey, 2019). On the other hand, BlastN analysis revealed that our Rictor siRNA sequence did not complement another gene sequence. In addition, we previously showed that inhibition of mTORC2 signaling by utilizing a single Rictor siRNA sequence in PHT cells did not alter mTORC1 signaling (Rosario et al., 2013), implying that Rictor silencing is highly specific in targeting mTORC2 signaling. Furthermore, we showed that co-transfection of Raptor siNA (to silence mTORC1 signaling) with DEPTOR siRNA (endogenous inhibitor of mTORC1 and mTORC2 signaling) to activate mTORC2 signaling in PHT cells. Notably, activating the mTORC2 signaling stimulated the PHT cell function, such as amino acid transport, which our Rictor siRNA decreases. These findings strongly suggest that the Rictor siRNA employed suppresses mTORC2 signaling specifically, with no substantial off-target effects.

## Conclusion

We demonstrate that mTORC2 signaling regulates the expression of trophoblast genes involved in pro-inflammatory, inflammatory, and micronutrient transport and angiogenesis, representing novel links between mTOR signaling and placental functions critical for normal fetal growth and development. Furthermore, because placental mTOR signaling is inhibited in IUGR and activated in fetal overgrowth, we propose regulating the placental transcriptome by mTOR signaling directly contributes to altered placental function and fetal growth in common pregnancy complications. Thus, our findings provide clues for the underlying etiology of IUGR, which ultimately could translate into a novel therapeutic approach.

## Data Availability Statement

The datasets presented in this study can be found in online repositories. The names of the repository/repositories and accession number(s) can be found in the article/Supplementary Material.

## Ethics Statement

The studies involving human participants were reviewed and approved by the University of Texas Health Science Center. The patients/participants provided their written informed consent to participate in this study.

## Author Contributions

FR, LC, TJ, MG, and TP contributed to the experiments’ conception and design and performed collection, analysis, and data interpretation. ACK and FR performed bioinformatics analysis. FR, ACK, TJ, and TP wrote the manuscript. All authors approved the final version of the manuscript.

## Conflict of Interest

The authors declare that the research was conducted in the absence of any commercial or financial relationships that could be construed as a potential conflict of interest.

## Publisher’s Note

All claims expressed in this article are solely those of the authors and do not necessarily represent those of their affiliated organizations, or those of the publisher, the editors and the reviewers. Any product that may be evaluated in this article, or claim that may be made by its manufacturer, is not guaranteed or endorsed by the publisher.

## Footnotes

1
https://www.qiagenbioinformatics.com/products/ingenuity-pathway-analysis
