# Supplementary material for: Mechanistic Target of Rapamycin Complex 2 Regulation of the Primary Human Trophoblast Cell Transcriptome
Source: Front Cell Dev Biol. 2021 Nov 4;9:670980. doi: 10.3389/fcell.2021.670980 (PMC8599300; doi:10.3389/fcell.2021.670980)
Supplement: Supplementary file 1 [file Data_Sheet_1.pdf]

Supplemental Table 1: List of DEGs in response to mTORC2 inhbition in PHT cells.

Gene ID	Control PHT cells	Rictor PHT cells	Rictor PHT cells SEM	Ratio	Direction	p-value	Gene Identifier	Other ID	UG Cluster	Locus Link	Chromosome
HTR1A	0.17821	0.344485	0.043805	1.12	Up	0.024912146	NM_000524	ILMN_1813690	Hs.247940	3350	5
HTR3C	0.546463	0.451749	0.023848	1.07	Down	0.035495135	NM_130770	ILMN_1739275	Hs.632579	170572	3
AFAP1	0.362592	0.579626	0.062212	1.16	Up	0.049573572	NM_198595	ILMN_1701998	Hs.529369	60312	4
ADAR	2.778323	3.19457	0.124081	1.33	Up	0.016930312	NM_015840	ILMN_2320964	Hs.12341	103	1
ALDH1A1	2.650712	1.865536	0.07953	1.72	Down	0.032422129	NM_000689	ILMN_2096372	Hs.76392	216	9
ALDH1A1	3.180163	2.472002	0.090017	1.63	Down	0.037505615	NM_000689	ILMN_1709348	Hs.76392	216	9
A1BG	0.62414	0.869142	0.015268	1.19	Up	0.03239658	NM_130786	ILMN_2055271	Hs.529161	1	19
ANKAR	0.459569	0.607385	0.025774	1.11	Up	0.03847966	NM_022353	ILMN_2047676	Hs.60772	150709	2
ASB3	0.328814	0.496901	0.045126	1.12	Up	0.019433163	NM_016115	ILMN_1699606	Hs.40763	51130	2
ANKRD10	2.454715	2.885607	0.11653	1.35	Up	0.027135671	NM_017664	ILMN_2115218	Hs.525163	55608	13
ANKRD17	0.169413	0.282102	0.013934	1.08	Up	0.026986435	NM_032217	ILMN_1787064	Hs.719092	26057	4
ALG10B	0.489161	0.287692	0.008706	1.15	Down	0.036350232	NM_001013620	ILMN_3251482	Hs.259305	144245	12
ATG4A	1.009975	1.243976	0.024327	1.18	Up	0.044638385	NM_178270	ILMN_2313782	Hs.8763	115201	X
ATRIP	0.745895	0.978102	0.033738	1.17	Up	0.020524391	NM_032166	ILMN_1653896	Hs.694840	84126	3
BRI3BP	0.609249	0.478725	0.045701	1.09	Down	0.041074345	NM_080626	ILMN_1797693	Hs.596464	140707	12
CELSR3	0.144348	0.315132	0.029149	1.13	Up	0.035068386	NM_001407	ILMN_1691290	Hs.631926	1951	3
CALCOCO2	0.903343	1.092205	0.038124	1.14	Up	0.006295104	NM_005831	ILMN_1755504	Hs.514920	10241	17
-	6.063775	6.339109	0.084454	1.21	Up	0.030279102	NR_003288	ILMN_2180519	-	-	-
CACNA1C	0.177076	0.350608	0.029225	1.13	Up	0.017157539	NM_000719	ILMN_1666775	Hs.118262	775	12
CHERP	1.140037	1.347524	0.059013	1.15	Up	0.014047411	NM_006387	ILMN_1798083	Hs.631627	10523	19
CAPS2	0.806531	0.946018	0.045359	1.1	Up	0.030329954	NM_032606	ILMN_3307742	Hs.407154	84698	12
CAST	0.573072	0.327905	0.050054	1.19	Down	0.012843796	NM_001042443	ILMN_1783627	Hs.436186	831	5

CMBL	0.387543	0.20972	0.032707	1.13	Down	0.042541699	NM_138809	ILMN_1709634	Hs.192586	134147	5
CPZ	1.378554	1.575299	0.029806	1.15	Up	0.026316981	NM_001014447	ILMN_1705258	Hs.78068	8532	4
CBL	0.575899	0.884828	0.082739	1.24	Up	0.035649693	NM_005188	ILMN_1716080	Hs.504096	867	11
CSNK1G3	0.704127	0.899536	0.037068	1.15	Up	0.007708085	NM_001031812	ILMN_1652024	Hs.129206	1456	5
CSNK2A1	2.105444	2.336212	0.075918	1.17	Up	0.033178454	NM_001895	ILMN_2386354	Hs.644056	1457	20
-	0.875158	1.114546	0.076546	1.18	Up	0.033328807	NR_002207	ILMN_1746375	-	283106	-
CASP9	1.072777	1.251665	0.057046	1.13	Up	0.028272848	NM_032996	ILMN_1718070	Hs.329502	842	1
CNOT1	0.5929	0.857573	0.080573	1.2	Up	0.019816515	NM_206999	ILMN_1682501	Hs.716474	23019	16
CD2AP	0.843019	1.079808	0.07424	1.18	Up	0.030415074	NM_012120	ILMN_1730433	Hs.485518	23607	6
CD80	0.435819	0.176548	0.047224	1.2	Down	0.012827183	NM_005191	ILMN_1716736	Hs.838	941	3
-	0.509255	0.631257	0.030981	1.09	Up	0.02372337	AK127458	ILMN_1896431	Hs.255813	-	16
-	4.386319	4.589018	0.021499	1.15	Up	0.040371548	NM_182905	ILMN_1655952	Hs.585931	376475	2
-	0.099738	0.298593	0.021256	1.15	Up	0.005834309	XM_001713703	ILMN_3247390	Hs.633109	100132347	X
CADM4	0.43541	0.617448	0.041512	1.13	Up	0.007385379	NM_145296	ILMN_1812096	Hs.370984	199731	19
CLN8	0.327268	0.077726	0.056741	1.19	Down	0.022232599	NM_018941	ILMN_1684576	Hs.127675	2055	8
CRCP	4.506378	4.815779	0.062072	1.24	Up	0.039329346	NM_014478	ILMN_2381537	Hs.300684	27297	7
-	0.38345	0.527226	0.030366	1.1	Up	0.010855603	NR_024337	ILMN_3241665	-	-	-
C1orf149	0.432126	0.544355	0.033511	1.08	Up	0.024135254	NM_022756	ILMN_2076940	Hs.17118	64769	1
-	0.654645	0.880262	0.057393	1.17	Up	0.01066802	XM_376965	ILMN_1773175	-	127003	-
C1orf2	2.609319	2.865442	0.06986	1.19	Up	0.03049507	NM_006589	ILMN_1795026	Hs.348308	10712	1
C1orf25	1.038575	1.155392	0.022442	1.08	Up	0.033573786	NM_030934	ILMN_1665300	Hs.107149	81627	1
C1orf91	0.521378	0.670288	0.042791	1.11	Up	0.02475409	NM_019118	ILMN_1673752	Hs.272299	56063	1
C12orf48	0.381372	0.173882	0.065521	1.15	Down	0.024319908	NM_017915	ILMN_1727055	Hs.330663	55010	12
C16orf70	1.066365	1.291397	0.057386	1.17	Up	0.016380231	NM_025187	ILMN_1711703	Hs.513666	80262	16
C17orf39	0.735081	0.943753	0.064816	1.16	Up	0.033521282	NM_024052	ILMN_1656427	Hs.187422	79018	17
C17orf63	1.847011	2.068323	0.047684	1.17	Up	0.006281841	NM_001077498	ILMN_1812441	Hs.564533	55731	17
C17orf85	0.675814	0.993757	0.062116	1.25	Up	0.030583611	NM_018553	ILMN_2221076	Hs.120963	55421	17
C17orf87	0.367455	0.124797	0.060398	1.18	Down	0.043944038	NM_207103	ILMN_1682761	Hs.462080	388325	17
-	0.628794	0.844901	0.009568	1.16	Up	0.018203763	NM_174947	ILMN_1730818	-	284424	-
C2orf57	0.704709	0.870927	0.038219	1.12	Up	0.03340606	NM_152614	ILMN_1768007	Hs.98104	165100	2
C2orf68	0.640033	0.47978	0.046518	1.12	Down	0.032246914	NM_001013649	ILMN_1679771	Hs.516159	388969	2
C2orf88	1.020807	0.741018	0.088131	1.21	Down	0.033196078	NM_032321	ILMN_1787526	Hs.389311	84281	2

C21orf66	0.506046	0.719719	0.031839	1.16	Up	0.006951017	NM_013329	ILMN_1712936	Hs.644004	94104	21
-	0.566347	0.731512	0.047274	1.12	Up	0.041664386	NR_003545	ILMN_1658439	-	-	-
C7orf49	1.742097	1.436448	0.041804	1.24	Down	0.006823457	NM_024033	ILMN_1740903	Hs.521213	78996	7
C9orf24	0.173229	0.34088	0.035853	1.12	Up	0.005469454	NM_147169	ILMN_1763695	Hs.50334	84688	9
C9orf3	0.253057	0.39134	0.032686	1.1	Up	0.010972475	NM_032823	ILMN_1674629	Hs.434253	84909	9
CMTM4	2.336165	2.018422	0.106435	1.25	Down	0.0370133	NM_181521	ILMN_1815319	Hs.643961	146223	16
CLRN1	0.370468	0.495591	0.04033	1.09	Up	0.043096769	NM_174878	ILMN_1741705	Hs.380222	7401	3
CC2D1B	0.336433	0.081162	0.058971	1.19	Down	0.048924787	NM_032449	ILMN_2149935	Hs.591451	200014	1
CCDC52	0.413769	0.604309	0.008337	1.14	Up	0.00068783	NM_144718	ILMN_1742380	Hs.477144	152185	3
COL6A2	0.242463	0.383676	0.030431	1.1	Up	0.00722639	NM_001849	ILMN_1809928	Hs.420269	1292	21
CPLX1	0.547951	0.676253	0.03982	1.09	Up	0.040072292	NM_006651	ILMN_1801703	Hs.478930	10815	4
CPLX2	0.435229	0.639805	0.052425	1.15	Up	0.025239894	NM_0010082<br>20	ILMN_1669382	Hs.193235	10814	5
CUL1	2.725488	2.952825	0.053655	1.17	Up	0.030087197	NM_003592	ILMN_1749629	Hs.146806	8454	7
CCBL1	0.384854	0.544881	0.03812	1.12	Up	0.037970655	NM_004059	ILMN_1764096	Hs.495250	883	9
-	1.964852	2.327935	0.08245	1.29	Up	0.007700224	NR_002174	ILMN_2086612	-	8418	-
CYP26A1	0.483414	0.638017	0.030819	1.11	Up	0.043683973	NM_057157	ILMN_1788131	Hs.150595	1592	10
DEPDC5	0.32008	0.506417	0.051036	1.14	Up	0.015319329	NM_0010071<br>88	ILMN_1800711	Hs.435022	9681	22
DMRTB1	0.604205	0.741093	0.032741	1.1	Up	0.035735748	NM_033067	ILMN_1754187	Hs.131654	63948	1
DFFA	1.667269	1.834082	0.043357	1.12	Up	0.044715126	NM_213566	ILMN_1667213	Hs.484782	1676	1
DOPEY2	0.541351	0.409907	0.011157	1.1	Down	0.012228844	NM_005128	ILMN_1741711	Hs.204575	9980	21
DBNL	1.909924	1.79406	0.023854	1.08	Down	0.048296225	NM_014063	ILMN_2376289	Hs.436500	28988	7
DYRK1A	1.582054	1.887916	0.087484	1.24	Up	0.044782418	NM_130437	ILMN_1660663	Hs.719269	1859	21
DNHD1	0.186005	0.325258	0.030562	1.1	Up	0.032369382	NM_144666	ILMN_1810267	Hs.377188	144132	11
EFEMP1	2.019757	1.824552	0.058296	1.14	Down	0.041311293	NM_004105	ILMN_1735877	Hs.76224	2202	2
EVPL	0.506469	0.71987	0.068274	1.16	Up	0.045792295	NM_001988	ILMN_1727288	Hs.500635	2125	17
EPB41L1	0.652831	0.935123	0.066372	1.22	Up	0.012550216	NM_012156	ILMN_1716507	Hs.716399	2036	20
EIF4A1	0.990628	1.256191	0.062382	1.2	Up	0.013823344	NR_002912	ILMN_3247018	Hs.129673	1973	17
EIF4EBP2	2.369635	2.683109	0.080473	1.24	Up	0.025787034	NM_004096	ILMN_1728083	Hs.695953	1979	10
-	0.18787	0.312581	0.021787	1.09	Up	0.005417359	NM_0010072<br>34	ILMN_1792107	-	1161	-
EXOC3L2	0.334236	0.484725	0.021711	1.11	Up	0.036503828	NM_138568	ILMN_1670038	Hs.337557	90332	19
FBXL20	1.847837	2.126721	0.058117	1.21	Up	0.049099042	NM_032875	ILMN_1754489	Hs.462946	84961	17
FBXO30	0.437135	0.656398	0.037104	1.16	Up	0.01091759	NM_032145	ILMN_2168992	Hs.421095	84085	6
FUBP1	0.359186	0.553474	0.048324	1.14	Up	0.010271111	NM_003902	ILMN_1776552	Hs.567380	8880	1

FAIM	0.258117	0.102036	0.033417	1.11	Down	0.023119555	NM_001033032	ILMN_2351548	Hs.173438	55179	3
FA2H	0.758598	0.911615	0.045503	1.11	Up	0.024300587	NM_024306	ILMN_1791531	Hs.461329	79152	16
FCF1	0.436939	0.60556	0.034345	1.12	Up	0.044145755	NM_015962	ILMN_2189869	Hs.579828	51077	14
FOXO3	3.240916	3.538892	0.095976	1.23	Up	0.025928833	NM_201559	ILMN_1681703	Hs.220950	2309	6
FPR1	0.790899	0.421052	0.035705	1.29	Down	0.014539782	NM_002029	ILMN_2092118	Hs.753	2357	19
-	0.14376	0.253446	0.016452	1.08	Up	0.007039562	NM_177442	ILMN_1735918	-	29960	-
GPRC5C	0.292651	0.417282	0.045534	1.09	Up	0.041468017	NM_022036	ILMN_1774528	Hs.446438	55890	17
GABBR1	0.133492	0.279193	0.025714	1.11	Up	0.028596941	NM_021903	ILMN_1658965	Hs.167017	2550	6
GGCT	1.847989	1.685257	0.031471	1.12	Down	0.025094833	NM_024051	ILMN_2101526	Hs.530024	79017	7
GFRA3	0.227188	0.355119	0.011257	1.09	Up	0.03516413	NM_001496	ILMN_1810716	Hs.58042	2676	5
GTF2B	2.332377	2.650301	0.070977	1.25	Up	0.018455964	NM_001514	ILMN_1737857	Hs.481852	2959	1
GINS3	0.628864	0.532932	0.014186	1.07	Down	0.007759912	NM_022770	ILMN_1754272	Hs.47125	64785	16
GLUL	0.738431	0.411831	0.045599	1.25	Down	0.000419319	NM_001033056	ILMN_1653496	Hs.518525	2752	1
CGA	7.06783	6.893127	0.027103	1.13	Down	0.00644687	NM_000735	ILMN_1734176	Hs.119689	1081	6
GP6	0.76003	0.950392	0.059272	1.14	Up	0.045843498	NM_001083899	ILMN_1732269	Hs.661752	51206	19
-	0.261354	0.44659	0.051389	1.14	Up	0.022571691	NR_002785	ILMN_3239955	-	-	-
GGA3	0.881901	1.136471	0.041761	1.19	Up	0.037677674	NM_138619	ILMN_1675982	Hs.87726	23163	17
GDF15	7.174531	6.99096	0.062378	1.14	Down	0.033286886	NM_004864	ILMN_2188862	Hs.616962	9518	19
-	0.399574	0.593463	0.071282	1.14	Up	0.045299637	NR_002164	ILMN_1746403	-	401375	-
GIMAP8	0.748037	0.221857	0.085445	1.44	Down	0.049542245	NM_175571	ILMN_1747305	Hs.647121	155038	7
GBP5	0.717148	0.124168	0.081755	1.51	Down	0.01506381	NM_052942	ILMN_2114568	Hs.513726	115362	1
-	1.204022	0.961171	0.06735	1.18	Down	0.019091395	NM_017939	ILMN_1763663	-	55027	-
HCST	0.687027	0.372215	0.092961	1.24	Down	0.048520102	NM_014266	ILMN_2396991	Hs.117339	10870	19
HPS3	0.618221	0.92276	0.076641	1.24	Up	0.020510022	NM_032383	ILMN_1762224	Hs.591311	84343	3
HHLA3	0.377351	0.512637	0.047549	1.1	Up	0.039235508	NM_001031693	ILMN_1802338	Hs.142245	11147	1
HNRNPK	4.346068	4.523818	0.063791	1.13	Up	0.045180836	NM_031263	ILMN_3179371	Hs.522257	3190	9
HIST1H2AC	2.75026	3.095308	0.092695	1.27	Up	0.017679158	NM_003512	ILMN_1792689	Hs.699831	8334	6
HIST1H2BD	3.651974	4.373395	0.091539	1.65	Up	0.015859226	NM_138720	ILMN_1651496	Hs.591797	3017	6
HIST1H2BD	2.465791	3.131308	0.100709	1.59	Up	0.015286937	NM_138720	ILMN_1758623	Hs.591797	3017	6
HIST1H3E	0.232064	0.422594	0.047346	1.14	Up	0.041181241	NM_003532	ILMN_3235949	Hs.443021	8353	6
HIST2H4A	0.804955	1.200249	0.028015	1.32	Up	0.045121215	NM_003548	ILMN_2115340	Hs.655235	8370	1
HIPK2	0.372606	0.171347	0.025004	1.15	Down	0.008964236	NM_022740	ILMN_1687440	Hs.397465	28996	7

HSCB	1.685765	1.942451	0.082175	1.19	Up	0.030305731	NM_172002	ILMN_2184789	Hs.632780	150274	22
HYDIN	0.329618	0.420552	0.021375	1.07	Up	0.006064051	NM_032821	ILMN_1654063	Hs.461229	54768	16
-	0.245088	0.167792	0.014493	1.06	Down	0.006085799	NM_001013708	ILMN_1664289	-	440570	-
LOC643100	0.302542	0.486221	0.035949	1.14	Up	0.010653755	XM_931316	ILMN_1692045	Hs.136333	643100	4
LOC727796	0.376261	0.271328	0.032352	1.08	Down	0.022850102	XR_015179	ILMN_3295838	Hs.647411	727796	17
-	0.096738	0.31225	0.058648	1.16	Up	0.015768766	XM_378549	ILMN_1815218	-	91948	-
LOC100128440	0.385922	0.135554	0.041025	1.19	Down	0.024446901	XM_001726725	ILMN_3191655	Hs.718548	100128440	17
LOC100128444	0.188932	0.385883	0.029072	1.15	Up	0.002805496	BM690686	ILMN_1845757	Hs.710979	100128444	7
LOC100129697	0.072481	0.286689	0.049834	1.16	Up	0.009024471	XM_001715229	ILMN_3263290	Hs.689377	100129697	16
LOC100129781	0.779299	1.059977	0.060783	1.21	Up	0.023956406	XM_001717065	ILMN_3273229	Hs.190748	100129781	16
LOC100129908	0.114652	0.300077	0.044602	1.14	Up	0.036988558	XM_001726037	ILMN_3190019	Hs.646997	100129908	6
-	6.114708	6.393301	0.053503	1.21	Up	0.004397694	NM_001039895	ILMN_2049343	-	-	-
IPO13	0.800118	0.983464	0.047755	1.14	Up	0.015865186	NM_014652	ILMN_1651229	Hs.158497	9670	1
IFITM1	1.818959	2.947224	0.338458	2.19	Up	0.034105749	NM_003641	ILMN_1801246	Hs.458414	8519	11
IFITM2	4.904708	5.434253	0.194093	1.44	Up	0.04819432	NM_006435	ILMN_1673352	Hs.709321	10581	11
IFRD2	1.122754	0.877759	0.046832	1.19	Down	0.013188631	NM_006764	ILMN_1742031	Hs.719200	7866	3
IL17REL	0.271933	0.522488	0.044031	1.19	Up	0.005057398	NM_001001694	ILMN_1722282	Hs.526712	400935	22
IL5RA	0.196283	0.36416	0.046443	1.12	Up	0.033395122	NM_000564	ILMN_1756455	Hs.68876	3568	3
IL6	0.055663	0.661489	0.205214	1.52	Up	0.030382732	NM_000600	ILMN_1699651	Hs.654458	3569	7
IL6ST	2.904873	3.382311	0.131373	1.39	Up	0.047088012	AB074172	ILMN_1849013	Hs.532082	3572	5
IL6ST	0.393284	0.538369	0.041922	1.11	Up	0.022826422	NM_002184	ILMN_1797861	Hs.532082	3572	5
INVS	0.689792	0.809816	0.013313	1.09	Up	0.026676076	NM_183245	ILMN_2360184	Hs.558477	27130	9
JMY	0.490271	0.6731	0.020757	1.14	Up	0.004896084	NM_152405	ILMN_1762080	Hs.482605	133746	5
KLK1	0.917414	1.139906	0.012374	1.17	Up	0.004006646	NM_002257	ILMN_1696450	Hs.123107	3816	19
KLK15	0.189781	0.42115	0.033051	1.17	Up	0.003462018	NM_017509	ILMN_2347097	Hs.567535	55554	19
KLHL29	0.946812	1.146414	0.050603	1.15	Up	0.036772803	XM_001717454	ILMN_3253471	Hs.130593	114818	2
KRTAP10-4	0.192692	0.325819	0.031535	1.1	Up	0.016855198	NM_198687	ILMN_1762711	Hs.567901	386672	21
KRTAP4-7	0.651614	0.786119	0.041832	1.1	Up	0.026599571	NM_033061	ILMN_1680432	Hs.712082	100132476	17
KIAA0100	0.838141	0.579594	0.062901	1.2	Down	0.022058891	NM_014680	ILMN_1718206	Hs.151761	9703	17
KIAA0323	2.258667	2.506902	0.062102	1.19	Up	0.009505331	NM_015299	ILMN_1654392	Hs.713590	23351	14

KIAA1632	0.362159	0.523267	0.018744	1.12	Up	0.012757781	NM_020964	ILMN_1838885	Hs.514843	57724	18
KIR2DL4	0.372549	0.447651	0.016788	1.05	Up	0.015231219	NM_002255	ILMN_1693207	Hs.661219	3805	19
KLRG1	0.239388	0.387903	0.04444	1.11	Up	0.049648877	NM_005810	ILMN_1658399	Hs.558446	10219	12
KNTC1	0.403381	0.540254	0.038651	1.1	Up	0.027277509	NM_014708	ILMN_1732516	Hs.300559	9735	12
LTF	0.533456	0.685906	0.034824	1.11	Up	0.012906063	NM_002343	ILMN_1677920	Hs.529517	4057	3
LCE3E	0.154003	0.26986	0.025929	1.08	Up	0.013348802	NM_178435	ILMN_1808220	Hs.490208	353145	1
LGALS3BP	2.223252	2.668265	0.115964	1.36	Up	0.01536681	NM_005567	ILMN_1659688	Hs.514535	3959	17
LEP	2.060373	2.357566	0.084361	1.23	Up	0.031676208	NM_000230	ILMN_2207505	Hs.194236	3952	7
LRFN1	0.384644	0.275313	0.041883	1.08	Down	0.044378214	NM_020862	ILMN_3247653	Hs.97860	57622	19
LRRC16B	0.543283	0.413653	0.030174	1.09	Down	0.016338452	NM_138360	ILMN_1777708	Hs.26135	90668	14
LRRC16B	0.660185	0.58568	0.02851	1.05	Down	0.049164403	NM_138360	ILMN_3244860	Hs.26135	90668	14
HLA-DQA2	0.218262	0.384237	0.028434	1.12	Up	0.021830143	NM_020056	ILMN_1680144	Hs.591798	3118	6
MASP1	0.274506	0.442623	0.030718	1.12	Up	0.016388979	NM_001031849	ILMN_1801996	Hs.89983	5648	3
MRGPRD	0.532005	0.646132	0.026519	1.08	Up	0.013977002	NM_198923	ILMN_1714980	Hs.527802	116512	11
MAML1	0.958683	1.128809	0.032023	1.13	Up	0.008462259	NM_014757	ILMN_1803060	Hs.631951	9794	5
MDM2	0.645741	0.905812	0.028973	1.2	Up	0.001579363	NM_002392	ILMN_1736829	Hs.484551	4193	12
MED15	0.179799	0.372535	0.052999	1.14	Up	0.030020293	NM_001003891	ILMN_1706200	Hs.517421	51586	22
MEIS2	0.709906	1.079328	0.104091	1.29	Up	0.049096725	NM_172315	ILMN_1695945	Hs.510989	4212	15
MAGEB2	0.170138	0.25681	0.018826	1.06	Up	0.011756813	NM_002364	ILMN_1688335	Hs.113824	4113	X
MREG	0.748005	0.568501	0.029802	1.13	Down	0.003492415	NM_018000	ILMN_1713679	Hs.707104	55686	2
MESDC1	0.69104	0.848093	0.045735	1.12	Up	0.042307827	NM_022566	ILMN_1781565	Hs.513071	59274	15
MESDC2	0.265059	0.136012	0.023817	1.09	Down	0.002443236	NM_015154	ILMN_1719797	Hs.578450	23184	15
-	0.19599	0.310713	0.024015	1.08	Up	0.008213396	NR_029907	ILMN_3308748	-	-	-
MDK	0.9124	1.235182	0.060927	1.25	Up	0.042938323	NM_001012334	ILMN_2349393	Hs.82045	4192	11
MCM8	5.42913	5.747354	0.110866	1.25	Up	0.044377932	NM_032485	ILMN_1798581	Hs.631506	84515	20
MRPS21	1.608097	1.813125	0.052518	1.15	Up	0.017891958	NM_018997	ILMN_1660292	Hs.405880	54460	1
MRPS27	1.859347	2.011904	0.0455	1.11	Up	0.03461134	NM_015084	ILMN_1711414	Hs.482491	23107	5
MAP3K5	0.860611	0.376192	0.065655	1.4	Down	0.036366826	NM_005923	ILMN_1726547	Hs.186486	4217	6
MAP4K3	1.415495	1.869282	0.073372	1.37	Up	0.024703716	NM_003618	ILMN_1813120	Hs.655750	8491	2
NAT12	0.462155	0.735758	0.094712	1.21	Up	0.043296957	NM_001011713	ILMN_2128087	Hs.165465	122830	14
NRD1	3.756253	3.940066	0.069169	1.14	Up	0.043695651	NM_002525	ILMN_1800897	Hs.584782	4898	1
-	0.47761	0.665315	0.030883	1.14	Up	0.011799898	NM_000907	ILMN_1681994	-	4882	-

NEDD9	0.435238	0.757989	0.056156	1.25	Up	0.032101702	NM_182966	ILMN_1743619	Hs.37982	4739	6
NBPF3	0.302688	0.17743	0.032146	1.09	Down	0.034550441	NM_032264	ILMN_1707244	Hs.325422	84224	1
NETO1	0.620744	0.764933	0.051141	1.11	Up	0.0429826	NM_138966	ILMN_1684081	Hs.465407	81832	18
NCF4	1.26734	0.88959	0.115531	1.3	Down	0.0412444	NM_000631	ILMN_2335704	Hs.474781	4689	22
-	0.238628	0.149746	0.017353	1.06	Down	0.040011966	NM_001031741	ILMN_1800445	-	152110	-
NSUN6	0.176367	0.353845	0.043008	1.13	Up	0.049430541	NM_182543	ILMN_1664646	Hs.396175	221078	10
NFIC	0.129082	0.298706	0.018147	1.12	Up	0.001997339	NM_005597	ILMN_1654942	Hs.170131	4782	19
NFKBIZ	2.392196	2.965222	0.157852	1.49	Up	0.030788975	NM_001005474	ILMN_1719695	Hs.319171	64332	3
NR1D1	0.794921	0.980232	0.060131	1.14	Up	0.037887149	NM_021724	ILMN_1708249	Hs.592130	9572	17
-	0.274101	0.487609	0.034141	1.16	Up	0.023631647	NR_002212	ILMN_3251356	-	440672	-
OR4L1	0.180726	0.397521	0.047573	1.16	Up	0.007644328	NM_001004717	ILMN_1722496	Hs.553574	122742	14
OR51I2	0.141968	0.455002	0.060889	1.24	Up	0.014413506	NM_001004754	ILMN_1700540	Hs.553733	390064	11
-	0.235287	0.458068	0.036592	1.17	Up	0.020192602	NR_002171	ILMN_2090351	-	283491	-
-	0.729524	0.940626	0.057699	1.16	Up	0.046281986	NM_001030011	ILMN_2382717	-	23596	-
OXNAD1	0.289892	0.141728	0.020567	1.11	Down	0.027160825	NM_138381	ILMN_1764770	Hs.655449	92106	3
OSBPL11	0.876592	1.098438	0.071014	1.17	Up	0.039274574	NM_022776	ILMN_1687410	Hs.477440	114885	3
PCTK3	0.392697	0.214518	0.049201	1.13	Down	0.023579291	NM_212503	ILMN_1784110	Hs.445402	5129	1
PDZD8	0.93809	1.076796	0.055038	1.1	Up	0.0497952	NM_173791	ILMN_1696962	Hs.501149	118987	10
PM20D1	0.232485	0.39365	0.053916	1.12	Up	0.028785342	NM_152491	ILMN_2293067	Hs.177744	148811	1
PHLPP	0.677192	0.576075	0.019338	1.07	Down	0.003443833	NM_194449	ILMN_1814661	Hs.465337	23239	18
PIK3R1	0.394657	0.265445	0.034722	1.09	Down	0.028563368	NM_181523	ILMN_1689057	Hs.132225	5295	5
PLA2G6	0.672825	0.859059	0.044664	1.14	Up	0.042459424	NM_001004426	ILMN_1697654	Hs.170479	8398	22
PLD1	0.72058	0.93812	0.057209	1.16	Up	0.043919961	NM_002662	ILMN_1719696	Hs.382865	5337	3
PLSCR1	1.076297	1.595537	0.206652	1.43	Up	0.04848712	NM_021105	ILMN_1745242	Hs.130759	5359	3
GART	0.971318	1.13681	0.052799	1.12	Up	0.046245038	NM_175085	ILMN_1793220	Hs.473648	2618	21
PTTG1	0.722181	0.408065	0.044236	1.24	Down	0.011211796	NM_004219	ILMN_2042771	Hs.350966	9232	5
PLAC8	1.056543	1.411924	0.10296	1.28	Up	0.047854922	NM_016619	ILMN_1653026	Hs.546392	51316	4
PLEKHM3	2.589571	3.094288	0.150884	1.42	Up	0.039907954	XM_001725073	ILMN_3215461	Hs.159188	389072	2
PARP2	0.514735	0.677299	0.025201	1.12	Up	0.010135158	NM_001042618	ILMN_2354237	Hs.409412	10038	14
PCBP3	0.458953	0.620785	0.043088	1.12	Up	0.03151641	NM_020528	ILMN_1687216	Hs.474049	54039	21
KCNC4	0.385437	0.241647	0.018292	1.1	Down	0.016886449	NM_004978	ILMN_1792382	Hs.153521	3749	1

PJA1	0.679156	0.951626	0.057899	1.21	Up	0.010192088	NM_145119	ILMN_1734810	Hs.522679	64219	X
-	0.100151	0.32408	0.053122	1.17	Up	0.011369766	XM_945357	ILMN_1689710	-	-	-
-	0.364369	0.500746	0.034984	1.1	Up	0.036471944	XR_017961	ILMN_1784408	-	-	-
-	0.243949	0.179317	0.023111	1.05	Down	0.049506187	XM_001133960	ILMN_1767573	-	-	-
-	0.355104	0.519951	0.019578	1.12	Up	0.02980392	XM_944791	ILMN_1787648	-	-	-
-	0.24359	0.508245	0.082935	1.2	Up	0.021448301	XM_001726935	ILMN_3181439	-	-	-
-	0.29833	0.181847	0.038581	1.08	Down	0.04706583	XM_930136	ILMN_1651259	-	-	-
-	0.251832	0.400073	0.026812	1.11	Up	0.022204454	XM_001127939	ILMN_1653760	-	-	-
-	0.377012	0.543739	0.045391	1.12	Up	0.047336652	XM_935633	ILMN_1663054	-	-	-
-	0.351184	0.225861	0.011682	1.09	Down	0.006437295	XM_001134250	ILMN_3296663	-	-	-
-	0.267801	-0.08011	0.10108	1.27	Down	0.025109382	XM_001128502	ILMN_3246658	-	-	-
-	2.521581	2.91465	0.13202	1.31	Up	0.03018482	XM_001722060	ILMN_3302499	-	-	-
-	0.622966	0.739861	0.015382	1.08	Up	0.033837386	XR_000536	ILMN_1668736	-	-	-
-	0.283894	0.46872	0.022594	1.14	Up	0.029889808	XR_039972	ILMN_3307134	-	-	-
-	0.418067	0.606337	0.041576	1.14	Up	0.020156922	XM_930545	ILMN_1794233	-	-	-
-	0.369865	0.467164	0.016291	1.07	Up	0.034808395	XM_001134053	ILMN_1855430	-	-	-
-	0.206328	0.272345	0.010204	1.05	Up	0.049439049	XM_944590	ILMN_1722916	-	-	-
-	0.501134	0.675935	0.062708	1.13	Up	0.039060078	XM_930648	ILMN_1670410	-	-	-
-	1.385205	1.57071	0.049323	1.14	Up	0.021443325	XM_936101	ILMN_1655694	-	-	-
-	0.087217	0.277852	0.064654	1.14	Up	0.041471844	XM_931123	ILMN_1712405	-	-	-
-	0.331019	0.19885	0.03203	1.1	Down	0.038040308	XM_927693	ILMN_1714399	-	-	-
-	0.224378	0.094838	0.015227	1.09	Down	0.00063824	XM_934553	ILMN_1778992	-	-	-
-	0.193912	0.444211	0.039114	1.19	Up	0.003340357	XM_937539	ILMN_1710641	-	-	-
-	0.650309	0.817364	0.056811	1.12	Up	0.034736443	XM_945688	ILMN_1698407	-	-	-
-	0.217232	0.339207	0.013898	1.09	Up	0.004343853	XM_940415	ILMN_1713128	-	-	-
-	0.293345	0.487287	0.04912	1.14	Up	0.021459646	XM_941669	ILMN_1762858	-	-	-
-	0.232715	0.426758	0.034188	1.14	Up	0.00337986	XM_001128891	ILMN_1658697	-	-	-
-	0.102016	0.392382	0.050858	1.22	Up	0.010225099	XM_933834	ILMN_1652982	-	-	-
-	1.287432	1.664007	0.099495	1.3	Up	0.01541425	XR_037866	ILMN_3187283	-	-	-
-	3.12399	3.569736	0.100462	1.36	Up	0.013193873	XR_038705	ILMN_3231550	-	-	-
-	1.01759	0.856299	0.056552	1.12	Down	0.048493356	XR_039693	ILMN_3199974	-	-	-

-	0.094681	0.276003	0.024278	1.13	Up	0.037671854	XR_039290	ILMN_3244476	-	-	-
-	1.518132	1.718192	0.073562	1.15	Up	0.04603137	XR_038632	ILMN_3294033	-	-	-
-	1.069417	1.293867	0.073009	1.17	Up	0.039041487	XR_038551	ILMN_3277715	-	-	-
-	0.179271	0.3043	0.042682	1.09	Up	0.047213956	XR_037389	ILMN_3286875	-	-	-
-	0.40958	0.632952	0.031025	1.17	Up	0.013631361	XR_039772	ILMN_3283244	-	-	-
-	3.028657	3.333417	0.087885	1.24	Up	0.047031017	XR_038497	ILMN_3278995	-	-	-
-	1.045462	1.232426	0.028585	1.14	Up	0.036358205	XR_017355	ILMN_3290261	-	-	-
-	0.625036	0.859596	0.058437	1.18	Up	0.019704934	XR_015272	ILMN_3209631	-	-	-
-	0.139328	0.304147	0.050888	1.12	Up	0.037885652	XR_037029	ILMN_3249452	-	-	-
-	0.54293	0.786897	0.038536	1.18	Up	0.004795811	XR_015979	ILMN_3230300	-	-	-
-	0.777555	0.938266	0.01638	1.12	Up	0.034509928	XM_945626	ILMN_1752028	-	-	-
-	0.254712	0.348789	0.027573	1.07	Up	0.048721175	XM_944909	ILMN_1740972	-	-	-
-	6.698273	6.484648	0.028407	1.16	Down	0.003116855	XM_939484	ILMN_3220792	-	-	-
-	0.242839	0.130598	0.029297	1.08	Down	0.034354034	XM_935348	ILMN_1773174	-	-	-
-	3.341924	3.048122	0.046479	1.23	Down	0.015981592	XR_017614	ILMN_1772888	-	-	-
-	0.750265	0.997487	0.045045	1.19	Up	0.002737339	XM_929542	ILMN_1660576	-	-	-
-	1.173502	1.404029	0.09093	1.17	Up	0.045970142	XM_940588	ILMN_1799699	-	-	-
-	0.332966	0.190213	0.024483	1.1	Down	0.03645912	XM_001127580	ILMN_1802761	-	-	-
-	2.431007	2.797964	0.113484	1.29	Up	0.046955489	XM_001723141	ILMN_3289262	-	-	-
-	0.211218	0.385407	0.034669	1.13	Up	0.025283251	XM_001717755	ILMN_3245093	-	-	-
-	0.740632	0.913372	0.044484	1.13	Up	0.046898369	XM_001724644	ILMN_3197732	-	-	-
-	0.17483	0.271736	0.03002	1.07	Up	0.034821511	XM_936822	ILMN_3200070	-	-	-
-	0.596128	0.420277	0.059064	1.13	Down	0.035585471	XM_943003	ILMN_1703369	-	-	-
-	0.326521	0.474091	0.026162	1.11	Up	0.014239076	XM_209824	ILMN_1790173	-	285929	-
-	0.185474	0.353916	0.022727	1.12	Up	0.034602887	XR_019367	ILMN_1809562	-	-	-
-	0.530297	0.65204	0.023075	1.09	Up	0.028084253	XM_001124642	ILMN_1694752	-	-	-
-	0.176132	0.385882	0.018518	1.16	Up	0.005292564	XM_001128785	ILMN_1653185	-	-	-
-	0.25162	0.137784	0.020061	1.08	Down	0.01732448	XM_937850	ILMN_1694327	-	-	-
-	0.361781	0.199391	0.022271	1.12	Down	0.012565406	XM_944469	ILMN_1695899	-	-	-
-	0.268071	0.207517	0.011293	1.04	Down	0.041510846	XM_001126197	ILMN_3301590	-	-	-
-	0.129253	0.291645	0.044084	1.12	Up	0.026291234	XM_942253	ILMN_1684977	-	-	-

-	0.525832	0.663176	0.035147	1.1	Up	0.01966927	XM_943679	ILMN_1718311	-	-	-
PPM1A	0.390386	0.664284	0.032251	1.21	Up	0.000898229	NM_177951	ILMN_1690077	Hs.130036	5494	14
PPM1D	3.106032	3.458758	0.084044	1.28	Up	0.018240839	NM_003620	ILMN_1670875	Hs.591184	8493	17
PRPF40A	0.094592	0.265308	0.037873	1.13	Up	0.040069913	NM_017892	ILMN_1666648	Hs.643580	55660	2
PUS7L	0.37186	0.228138	0.019013	1.1	Down	0.029233899	NM_0010986 15	ILMN_1747184	Hs.445814	83448	12
PSORS1C2	0.231891	0.113409	0.020614	1.09	Down	0.020026678	NM_014069	ILMN_1653447	Hs.146824	170680	6
PNPO	2.3759	1.954534	0.084824	1.34	Down	0.049129736	NM_018129	ILMN_1684289	Hs.631742	55163	17
RABGAP1L	0.534061	0.300164	0.020584	1.18	Down	0.006711207	NM_0010352 30	ILMN_1708721	Hs.585378	9910	1
RAB28	0.623969	0.715842	0.022327	1.07	Up	0.019160388	NM_0010179 79	ILMN_2293992	Hs.656060	9364	4
-	0.298231	0.408119	0.021746	1.08	Up	0.026030072	NM_052949	ILMN_1688218	-	115727	-
RHOF	0.247813	0.406692	0.023917	1.12	Up	0.037506699	NM_019034	ILMN_1652918	Hs.524804	54509	12
RASAL3	0.178735	0.307498	0.015572	1.09	Up	0.046944199	NM_022904	ILMN_3238803	Hs.136979	64926	19
RASL10B	0.203338	0.320232	0.022998	1.08	Up	0.023602997	NM_033315	ILMN_1788813	Hs.437035	91608	17
RCE1	2.417887	2.704469	0.088489	1.22	Up	0.02525684	NM_0010322 79	ILMN_1685002	Hs.654972	9986	11
RNLS	0.086315	0.291281	0.021287	1.15	Up	0.008332022	NM_018363	ILMN_1783873	Hs.149849	55328	10
RFC3	0.139857	0.261993	0.037116	1.09	Up	0.038061758	NM_181558	ILMN_1711381	Hs.115474	5983	13
RFC4	1.217727	1.035631	0.060548	1.13	Down	0.041469033	NM_181573	ILMN_1724489	Hs.714318	5984	3
RPA1	1.762588	1.943688	0.015706	1.13	Up	0.020527163	NM_002945	ILMN_1795719	Hs.461925	6117	17
RCOR2	0.236187	0.095467	0.029667	1.1	Down	0.016575743	NM_173587	ILMN_1791366	Hs.98788	283248	11
REXO1	1.783299	2.068939	0.048404	1.22	Up	0.003919041	NM_020695	ILMN_1753008	Hs.192477	57455	19
ARHGEF17	0.325633	0.477365	0.036644	1.11	Up	0.019579701	NM_014786	ILMN_1754562	Hs.533719	9828	11
RHBDL3	0.447175	0.306468	0.019616	1.1	Down	0.0026863	NM_138328	ILMN_1716019	Hs.655027	162494	17
-	5.330086	5.191549	0.04273	1.1	Down	0.044799682	XM_372926	ILMN_3293367	-	391370	-
-	0.287108	0.138713	0.021288	1.11	Down	0.008956357	XM_375543	ILMN_1775473	-	400652	-
RNF4	1.439565	1.77333	0.037205	1.26	Up	0.002397184	NM_002938	ILMN_1687941	Hs.66394	6047	4
RMI1	1.066281	1.277903	0.063748	1.16	Up	0.038338269	NM_024945	ILMN_1754051	Hs.284137	80010	9
RBM4	1.296622	1.556659	0.049178	1.2	Up	0.006867371	NM_002896	ILMN_1757439	Hs.533712	5936	11
RBM4	2.355001	2.605572	0.08981	1.19	Up	0.034950499	NM_002896	ILMN_1709042	Hs.533712	5936	11
RUFY2	0.277291	0.441548	0.023963	1.12	Up	0.032698705	NM_017987	ILMN_1707872	Hs.653144	55680	10
S100A8	1.786392	1.057368	0.199921	1.66	Down	0.016049513	NM_002964	ILMN_1729801	Hs.416073	6279	1
S100PBP	1.629131	1.86959	0.070848	1.18	Up	0.044287577	NM_022753	ILMN_2294274	Hs.440880	64766	1
SAFB	2.811944	3.220896	0.116826	1.33	Up	0.020669927	NM_002967	ILMN_1722059	Hs.23978	6294	19

SOST	0.172792	0.323115	0.025836	1.11	Up	0.048996086	NM_025237	ILMN_1781242	Hs.349204	50964	17
SPP1	6.345564	5.574901	0.160818	1.71	Down	0.008705769	NM_000582	ILMN_1651354	Hs.313	6696	4
SPP1	5.979136	5.307023	0.196205	1.59	Down	0.037747749	NM_001040058	ILMN_2374449	Hs.313	6696	4
SEPN1	1.795628	1.518806	0.058613	1.21	Down	0.039665907	NM_206926	ILMN_1760890	Hs.568578	57190	1
SPINK9	0.142831	0.370741	0.071775	1.17	Up	0.032583707	NM_001040433	ILMN_2045351	Hs.631798	643394	5
SETD8	1.297859	1.531423	0.056225	1.18	Up	0.03674827	NM_020382	ILMN_1651936	Hs.443735	387893	12
SH2D1B	0.198256	0.342295	0.032759	1.1	Up	0.02092871	NM_053282	ILMN_1701237	Hs.350581	117157	1
SH2B1	0.875331	1.06723	0.040469	1.14	Up	0.005097792	NM_015503	ILMN_2061185	Hs.15744	25970	16
SIRPB1	0.354156	0.460496	0.028204	1.08	Up	0.043067457	XR_018642	ILMN_1742442	Hs.710882	10326	20
-	1.821686	1.94373	0.030796	1.09	Up	0.043399577	NM_138781	ILMN_1667330	-	113386	-
LOC729595	0.632508	0.441103	0.055229	1.14	Down	0.024993424	XM_001130734	ILMN_3297398	Hs.659876	729595	5
LOC100132620	0.356087	0.576569	0.019335	1.17	Up	0.00983514	XM_001724221	ILMN_3246585	Hs.718878	100132620	16
LOC727773	0.241468	0.11376	0.02752	1.09	Down	0.033503911	XM_001126273	ILMN_1655093	Hs.529172	727773	2
-	0.285783	0.431706	0.051531	1.11	Up	0.040248959	XM_376822	ILMN_1767980	-	401497	-
LOC729264	0.979268	1.249042	0.079863	1.21	Up	0.036586834	XM_001133677	ILMN_1744252	Hs.513537	729264	16
SNIP1	1.31916	1.578918	0.091113	1.2	Up	0.039412709	NM_024700	ILMN_1651278	Hs.471951	79753	1
SCN1B	0.222586	0.408951	0.065957	1.14	Up	0.037677161	NM_199037	ILMN_1767666	Hs.436646	6324	19
SCN3B	0.72551	0.92456	0.058468	1.15	Up	0.031224579	NM_018400	ILMN_2412822	Hs.4865	55800	11
SLC13A1	0.265788	0.42005	0.026467	1.11	Up	0.003406535	NM_022444	ILMN_2204430	Hs.489849	6561	7
SLC14A1	0.237525	0.376318	0.017717	1.1	Up	0.004127317	NM_015865	ILMN_1805561	Hs.101307	6563	18
SLC19A2	0.80744	1.052926	0.029279	1.19	Up	0.01184436	NM_006996	ILMN_2201668	Hs.30246	10560	1
SLC25A29	0.237797	0.104075	0.033724	1.1	Down	0.03643146	NM_001039355	ILMN_2350801	Hs.578109	123096	14
SLC5A8	1.833749	2.218263	0.107846	1.31	Up	0.015296672	NM_145913	ILMN_1811221	Hs.444536	160728	12
SLC5A6	5.659733	5.449758	0.047067	1.16	Down	0.008564158	NM_021095	ILMN_1741054	Hs.435735	8884	2
SLC8A1	0.32909	0.444958	0.014153	1.08	Up	0.018828474	NM_021097	ILMN_1699520	Hs.468274	6546	2
SPEN	2.995294	3.303524	0.092894	1.24	Up	0.035616802	NM_015001	ILMN_1802611	Hs.558463	23013	1
SPATA18	0.158663	0.258681	0.013925	1.07	Up	0.011724904	NM_145263	ILMN_1667948	Hs.527090	132671	4
SMOX	0.132981	0.26411	0.029937	1.1	Up	0.03164882	NM_175842	ILMN_2280707	Hs.433337	54498	20
SFPQ	1.322131	1.15374	0.012045	1.12	Down	0.042348733	NM_005066	ILMN_1769931	Hs.355934	6421	1
SFRS18	1.644472	1.98472	0.103892	1.27	Up	0.038893997	NM_015491	ILMN_3299558	Hs.520287	25957	6
SFRS18	1.676814	1.93114	0.057466	1.19	Up	0.022098887	NM_032870	ILMN_2161357	Hs.520287	25957	6

SPRYD3	1.260215	1.487916	0.030439	1.17	Up	0.047717317	NM_032840	ILMN_1788095	Hs.343334	84926	12
ST3GAL2	0.267336	0.145124	0.037198	1.09	Down	0.036580601	NM_006927	ILMN_1714165	Hs.368611	6483	16
STAG1	1.738216	1.923643	0.041436	1.14	Up	0.046030521	NM_005862	ILMN_2151048	Hs.412586	10274	3
TAOK3	1.503728	1.716543	0.073102	1.16	Up	0.043379006	NM_016281	ILMN_3307863	Hs.644420	51347	12
TERF2IP	2.347839	2.527174	0.034945	1.13	Up	0.026429521	NM_018975	ILMN_1657983	Hs.301419	54386	16
TES	0.141171	0.303142	0.02834	1.12	Up	0.038930205	NM_152829	ILMN_2311798	Hs.592286	26136	7
TGIF1	3.748833	4.129647	0.099386	1.3	Up	0.044925688	NM_170695	ILMN_2318638	Hs.373550	7050	18
TK2	0.427286	0.298124	0.026358	1.09	Down	0.043979221	NM_004614	ILMN_1766814	Hs.512619	7084	16
TYMP	1.097137	0.66883	0.099598	1.35	Down	0.039440693	NM_0011137<br>56	ILMN_3223126	Hs.592212	1890	22
TYMP	0.862472	0.465011	0.089562	1.32	Down	0.024501834	NM_0011137<br>55	ILMN_3297126	Hs.592212	1890	22
TRAPPC6A	2.289384	2.090749	0.043893	1.15	Down	0.028282795	NM_024108	ILMN_1775703	Hs.466929	79090	19
-	0.177457	0.420701	0.064306	1.18	Up	0.03265393	DB302602	ILMN_1888264	Hs.579479	-	18
-	0.97726	0.761032	0.063545	1.16	Down	0.030936864	W25998	ILMN_1835017	Hs.633892	-	1
-	0.798115	0.585575	0.056908	1.16	Down	0.048245744	AI557007	ILMN_1908989	Hs.536748	-	10
-	0.110943	0.31512	0.015361	1.15	Up	0.0019693	AW297072	ILMN_1896737	Hs.543863	-	5
-	0.168652	0.369773	0.026357	1.15	Up	0.00051126	BQ019141	ILMN_1904111	Hs.619548	-	X
-	0.471862	0.296949	0.040144	1.13	Down	0.039367318	AW972698	ILMN_1895042	Hs.496730	-	X
-	0.242109	0.38243	0.032223	1.1	Up	0.007561134	BX118075	ILMN_1866314	Hs.130455	-	7
-	0.185424	0.323129	0.033119	1.1	Up	0.029379551	BX100285	ILMN_1848906	Hs.208715	-	9
-	0.197086	0.318846	0.031947	1.09	Up	0.020012352	BI757437	ILMN_1848499	Hs.156256	-	19
-	0.190943	0.306062	0.01206	1.08	Up	0.037083615	DB297053	ILMN_1861230	Hs.580105	-	2
-	0.272548	0.202978	0.022475	1.05	Down	0.038474623	BG547692	ILMN_1883050	Hs.186848	-	5
TLE1	2.470155	2.686583	0.034776	1.16	Up	0.038748907	NM_005077	ILMN_1751572	Hs.197320	7088	9
-	0.244639	0.428551	0.025966	1.14	Up	0.012287459	NM_0010011<br>88	ILMN_1743987	-	7226	-
TMEM216	1.238721	1.491462	0.076525	1.19	Up	0.03829488	NM_016499	ILMN_1732577	Hs.26745	51259	11
TMEM41A	0.990438	1.157524	0.046932	1.12	Up	0.037746532	NM_080652	ILMN_1685602	Hs.715632	90407	3
TMEM88	0.40465	0.520746	0.029684	1.08	Up	0.020088664	NM_203411	ILMN_1757129	Hs.389669	92162	17
TRIM14	0.282395	0.382198	0.018294	1.07	Up	0.030929233	NM_014788	ILMN_1713542	Hs.575631	9830	9
TRIM23	0.466	0.542303	0.013791	1.05	Up	0.013019355	NM_033227	ILMN_2327780	Hs.792	373	5
WRB	1.34676	1.113137	0.072519	1.18	Down	0.049218084	NM_004627	ILMN_1695092	Hs.198308	7485	21
TSC22D2	2.239088	2.701863	0.13866	1.38	Up	0.042324781	NM_014779	ILMN_2124187	Hs.715600	9819	3
TBCE	0.806887	0.920554	0.042135	1.08	Up	0.040840926	NM_003193	ILMN_2359014	Hs.498143	6905	1
TTLL4	0.574898	0.790966	0.039597	1.16	Up	0.005515131	NM_014640	ILMN_1746846	Hs.471405	9654	2

TUBB2B	0.030933	0.256752	0.033334	1.17	Up	0.001017295	NM_178012	ILMN_1680874	Hs.300701	347733	6
TFIP11	0.772211	1.177876	0.12862	1.32	Up	0.041029352	NM_001008697	ILMN_1695000	Hs.20225	24144	22
TWF1	0.577995	0.850249	0.079626	1.21	Up	0.028906888	NM_002822	ILMN_1681203	Hs.189075	5756	12
TYRO3	0.297106	0.211141	0.021702	1.06	Down	0.015735901	NM_006293	ILMN_1740169	Hs.381282	7301	15
UBQLNL	0.313652	0.513805	0.036907	1.15	Up	0.006046134	NM_145053	ILMN_1748907	Hs.10688	143630	11
UBR5	1.173116	1.365686	0.044028	1.14	Up	0.011702774	NM_015902	ILMN_2204726	Hs.591856	51366	8
USP21	0.768223	0.903304	0.043258	1.1	Up	0.030809862	NM_012475	ILMN_2349006	Hs.8015	27005	1
B3GNT1	0.653378	0.485181	0.050788	1.12	Down	0.039806116	NM_006876	ILMN_1715384	Hs.8526	11041	11
ULK3	1.029984	1.196049	0.04527	1.12	Up	0.031269289	NM_001099436	ILMN_1679495	Hs.513034	25989	15
UBTF	0.570779	0.761145	0.059984	1.14	Up	0.034255374	NM_001076683	ILMN_1806946	Hs.89781	7343	17
VPS4A	1.44031	1.655387	0.032875	1.16	Up	0.008783028	NM_013245	ILMN_1708946	Hs.128420	27183	16
VEGFA	0.635075	0.793203	0.041876	1.12	Up	0.021684861	NM_001025367	ILMN_1803882	Hs.73793	7422	6
-	0.460616	0.615276	0.039068	1.11	Up	0.019295678	NM_201994	ILMN_1664175	-	8674	-
WDR20	0.427324	0.605893	0.03886	1.13	Up	0.018591648	NM_144574	ILMN_2361324	Hs.36859	91833	14
WDR44	0.260637	0.407799	0.010684	1.11	Up	0.001659377	NM_019045	ILMN_2178618	Hs.98510	54521	X
WWP2	0.89754	1.069395	0.049342	1.13	Up	0.044482487	NM_199424	ILMN_1668847	Hs.408458	11060	16
ZADH2	0.645441	0.545088	0.024524	1.07	Down	0.037282137	NM_175907	ILMN_1795063	Hs.465433	284273	18
ZSCAN2	0.454666	0.643982	0.03695	1.14	Up	0.008071691	NM_001007072	ILMN_1653163	Hs.594023	54993	15
ZC3H14	1.291558	1.503226	0.048847	1.16	Up	0.01292924	NM_207660	ILMN_2335669	Hs.325846	79882	14
ZC3H14	0.445904	0.243592	0.030409	1.15	Down	0.007803602	NM_024824	ILMN_1714805	Hs.325846	79882	14
ZNF674	7.247913	7.023403	0.074114	1.17	Down	0.034495202	NM_001039891	ILMN_3237839	Hs.675818	641339	X
ZNF200	0.801267	0.977679	0.038677	1.13	Up	0.044994883	NM_198088	ILMN_2376833	Hs.632222	7752	16
ZNF3	0.061439	0.291516	0.072282	1.17	Up	0.04039595	NM_032924	ILMN_1778560	Hs.435302	7551	7
ZNF350	0.500819	0.738314	0.078253	1.18	Up	0.024182672	NM_021632	ILMN_1755850	Hs.407694	59348	19
ZNF407	0.14702	0.279977	0.033909	1.1	Up	0.048059737	NM_017757	ILMN_1753856	Hs.536490	55628	18
ZNF416	0.312257	0.399074	0.018507	1.06	Up	0.029976223	NM_017879	ILMN_1767811	Hs.247711	55659	19
ZNF470	0.843035	1.037948	0.044895	1.14	Up	0.037476831	NM_001001668	ILMN_1690863	Hs.204449	388566	19
-	0.395974	0.641385	0.031074	1.19	Up	0.000409346	XM_375646	ILMN_1748432	-	170958	-
ZNF566	0.272807	0.196329	0.022996	1.05	Down	0.029774442	NM_032838	ILMN_2125880	Hs.533939	84924	19
ZNF567	0.300164	0.600397	0.048466	1.23	Up	0.006574321	NM_152603	ILMN_1687544	Hs.412517	163081	19
ZNF57	0.239024	0.389864	0.027551	1.11	Up	0.005109432	NM_173480	ILMN_1691333	Hs.591378	126295	19

ZNF695	0.557246	0.411938	0.021553	1.11	Down	0.009676865	NM_020394	ILMN_3307683	Hs.669893	57116	1
ZNF827	1.385509	1.589204	0.055946	1.15	Up	0.017523338	NM_178835	ILMN_1727574	Hs.133916	152485	4
ZNF830	0.257046	0.424706	0.0499	1.12	Up	0.041026765	NM_052857	ILMN_3247592	Hs.413678	91603	17
ZCCHC11	0.585445	0.793519	0.051377	1.16	Up	0.038366784	NM_015269	ILMN_2373099	Hs.655407	23318	1
ZCCHC3	1.482434	1.729515	0.060947	1.19	Up	0.024426454	NM_033089	ILMN_1786852	Hs.28608	85364	20
ZDHHC16	4.322713	3.947294	0.045827	1.3	Down	0.001085889	NM_198045	ILMN_1763568	Hs.76662	84287	10
ZSWIM4	2.413293	2.912582	0.115017	1.41	Up	0.018006802	NM_023072	ILMN_2150654	Hs.466015	65249	19
ZNRD1	0.355276	0.493932	0.045604	1.1	Up	0.044569941	NM_170783	ILMN_1722894	Hs.57813	30834	6
	Up Regulated:										
	Down Regulated:										
