# Supplementary material for: Mechanistic Target of Rapamycin Complex 2 Regulation of the Primary Human Trophoblast Cell Transcriptome
Source: Front Cell Dev Biol. 2021 Nov 4;9:670980. doi: 10.3389/fcell.2021.670980 (PMC8599300; doi:10.3389/fcell.2021.670980)
Supplement: Supplementary file 2 [file Data_Sheet_2.pdf]

Materials and Methods:

Pathway analysis

The statistical enrichment of differentially expressed genes in pathways was calculated using the following formula: z-score = (r-n(R/N))/SQRT(n(R/N)(1-(R/N))(1-(n-1/N-1)); where R = total number of genes meeting selection criteria, N = total number of genes measured, r = number of genes meeting selection criteria with the specified GO term, and n = total number of genes measured with the specific GO term. Pathway enrichment analysis of differentially expressed genes has been used to reveal pathways impacted in biological systems (1). KEGG is a comprehensive knowledge base for assisting in the biological interpretation of large-scale molecular datasets. Using KEGG pathway enrichment analysis provides a way to filter gene expression data statistically within annotated biological systems (2). Calculations were performed using GeneSifter software default settings without user input. Pathways were considered significantly different between groups if the z-score for that pathway was greater than 2.0 or less than- 2.0 (3). Biological pathways were mapped by using the online tool KEGG (http://www.genome.jp/kegg/; Kyoto encyclopedia for genes and genomes) (2, 4-6)

As a first step in our discovery approach, we used a simple p-value < 0.05 cutoff to identify differential gene expression. Because all transcripts are not equal in terms of fold-change required to impact phenotype, we did not apply a fold-change cutoff. Furthermore, the Illumina BeadChip gene array includes an average of 30 probes per gene. Gene array data were first filtered in Genome Studio (Illumina) to eliminate gene intensities below background, which is defined by negative controls included on the BeadChip. All genes that passed background filter were then filtered by the detection p-value, which takes into account the signal quality of all probes for each gene (~30 probes). We used a 0.95 as a cutoff. This is standard practice as defined by Illumina for their arrays. Our second step in the discovery phase included stringent statistical filtering where we required significant enrichment of genes in pathways and networks. Pathways and networks that pass this statistical filter were used to inform specific hypotheses that can be tested. In this study, KEGG pathway analyses suggested that VEGF and Jak-STAT signaling pathways were upregulated in response to rictor silencing. Subsequent experiments in this study were then directed at validating the expression of some of the individual genes of KEGG pathways. Using Western blot, we have found that the protein expression of leptin, VEGF-A and IL-6 (genes belong to VEGF and Jak-STAT signaling pathway) were upregulated in PHT cells silenced with rictor siRNA.

String Analysis

The differentially expressed gene list was submitted into String database version 11.5 to obtain a protein-protein interaction network (7). The minimum required interaction score is 0.9 (highest confidence score). STRING analysis showed that among the significantly decreased DEGs, the PIK3R1(Phosphatidylinositol 3-kinase regulatory subunit alpha) gene interacts with PDPK1, TYRO3, HCST, MAP3K5, PTTG1, and CD81 (Supplemental Figure 2). Thus, this interaction depicts the importance of the PIK3R1 gene in this output. Conversely, STRING analysis for the significantly increased DEGs, the IL6, VEGFA, LEP, and FOXO9 genes had a more significant interaction network with other genes (Supplemental Figure 3).

Western blot Analysis

Western blot analysis was performed, as previously described (8). In brief, 5-10μg (placental homogenates) or 5 μg PHT cell lysates of total protein were loaded onto a NuPAGE Novex (Invitrogen) precast 4%–12% Bis-Tris gels. Electrophoresis was performed at a constant voltage of 200 V for 40 min. Proteins were transferred to nitrocellulose membranes at a constant 40 V. After transfer; membranes were blocked in 5% milk in Tris-buffered saline plus 0.1% Tween-20 for 1 h at room temperature. Membranes were incubated in primary antibodies (Anti-VEGF-A, Catalogue # ab214424, Primary ab dilution: 1:1K, Company: Abcam, Cambridge, United Kingdom; Anti-Leptin, Catalogue # ab219260, Primary ab dilution: 1:2K, Company: Abcam, Cambridge, United Kingdom; Anti-IL6, Catalogue # ab259341, Primary ab dilution: 1:1K, Company: Abcam, Cambridge, United Kingdom; Anti-osteopontin, Catalogue # ab166709, Primary ab dilution: 1:1K, Company: Abcam, Cambridge, United Kingdom; Anti-osteopontin, Catalogue # ab166709, Primary ab dilution: 1:1K, Company: Abcam, Cambridge, United Kingdom; Anti- SLC5A6, Catalogue # ab137214, Primary ab dilution: 1:1K, Company: Abcam, Cambridge, United Kingdom; Anti-Caspase-3 antibody, Catalogue # ab90437, Primary ab dilution: 1:3K, Company: Abcam, Cambridge, United Kingdom; Anti-Rictor, Catalogue # ab219950, Primary ab dilution: 1:1K, Company: Abcam, Cambridge, United Kingdom; Anti-Akt-Ser-473, Catalogue # 1C10B8, Primary ab dilution: 1:1K, Company: Thermo scientific, Massachusetts, US) overnight at 4°C and followed by incubation in corresponding secondary antibody for 1 h at room temperature. After washing, bands were visualized using ECL detection reagents (Pierce Biotechnology) and images obtained by G: Box Chemi system (Syngene). Target band densities were adjusted to loading by using Ponceau Red as a control. Blots were analyzed by using ImageJ software (National Institutes of Health: imagej.nih.gov/ij/). To minimize the introduction of experimental bias and variability across the gels, in each gel, we have loaded pooled placental homogenate sample and normalize the other conditions to the pooled sample value.

Pseudogene analysis

The pseudogenes listed in supplemental Table 1 were analyzed separately. First, the gene ID conversion tool (DAVID Bioinformatics Resources 6.8, NIAID/NIH) converted the pseudogene id (input: gene identifier) to gene id. Finally, separate Network analyses (ShinyGO v0.66: Gene Ontology Enrichment Analysis) for up-regulated and down-regulated genes were performed. Network analysis (Supplemental Figure 8) revealed that down-regulated pseudogenes were mainly involved in the regulation of defenses response, regulation of receptor cycling, mesenchyme development, and mesoderm development. Conversely, we did not find significant network enrichment for up-regulated pseudogenes. However, Gene Ontology (GO)- biological process (ShinyGO v0.66: Gene Ontology Enrichment Analysis) revealed that up-regulated pseudogenes were mainly involved in biological processes, including response to external stimulus (OPN3, PSPC1, RASGRP4, ERCC8), cellular component biogenesis (VAMP4, HSPA4, MRM2, PIP4K2B) and regulation of response to stimulus (ERCC8, PSPC1, RASGRP4, PIP4K2B).

Supplemental Figure 1. The study design showing the time point of siRNA mediated silencing of mTORC2 signaling and measurements of various parameters in PHT cells.

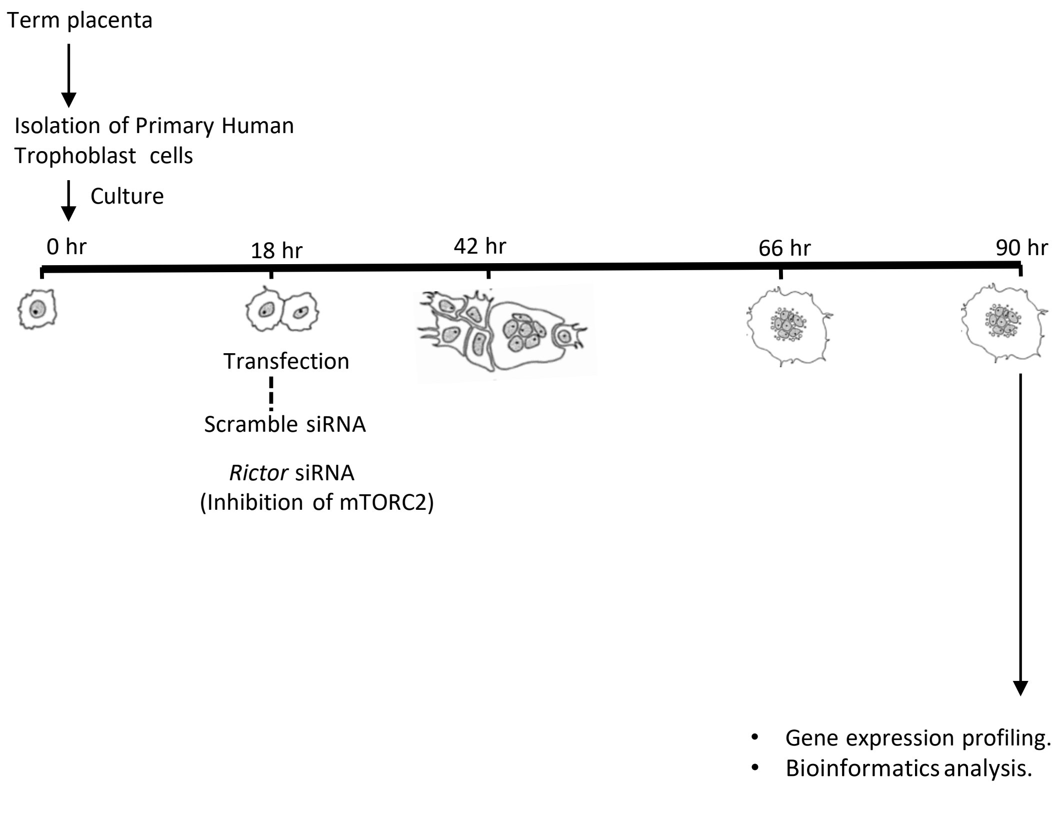

Supplemental Figure 2: STRING analysis output interaction network for differentially upregulated genes in rictor silenced PHT cells.

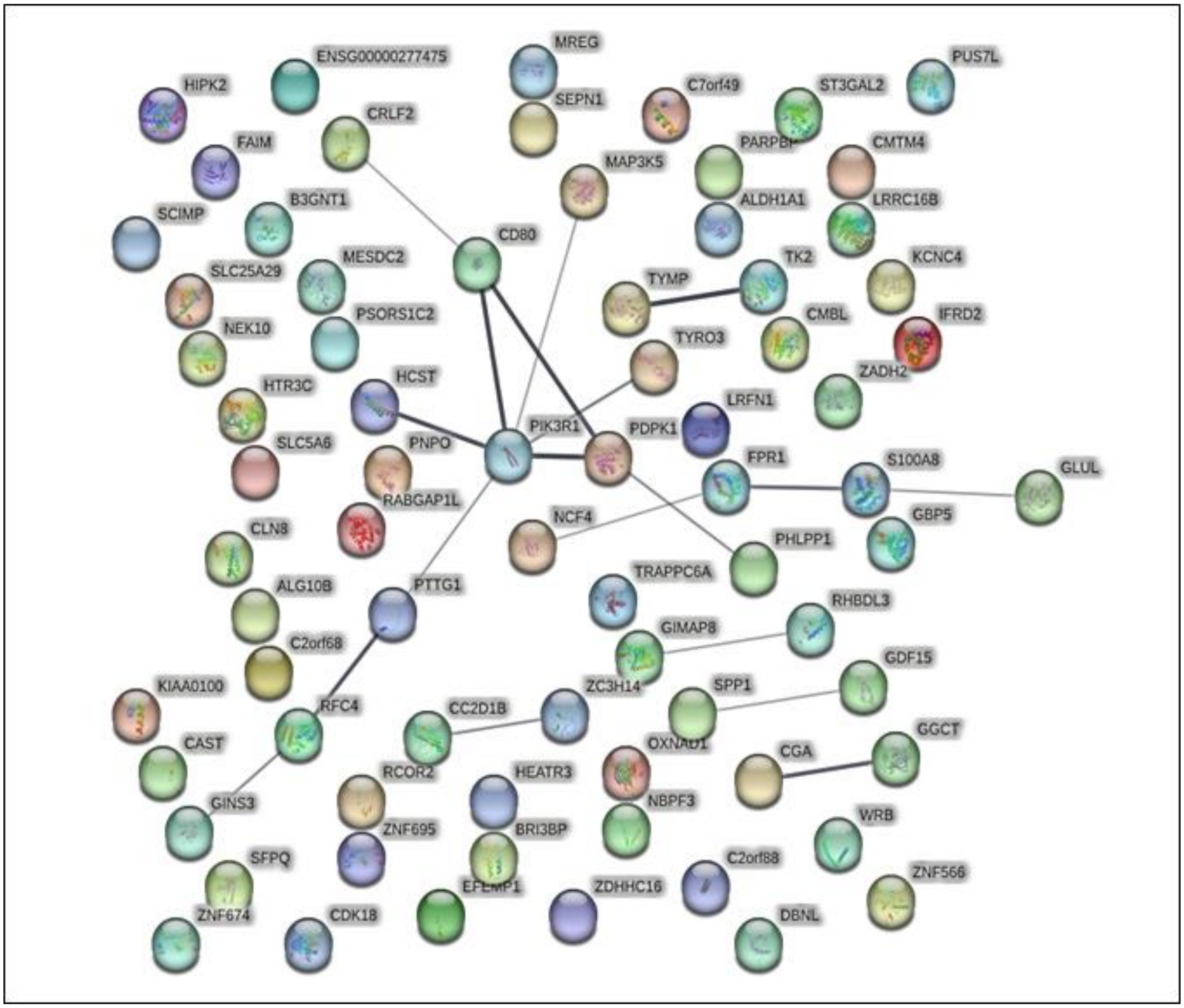

Supplemental Figure 3: STRING analysis output interaction network for differentially down regulated genes in rictor silenced PHT cells.

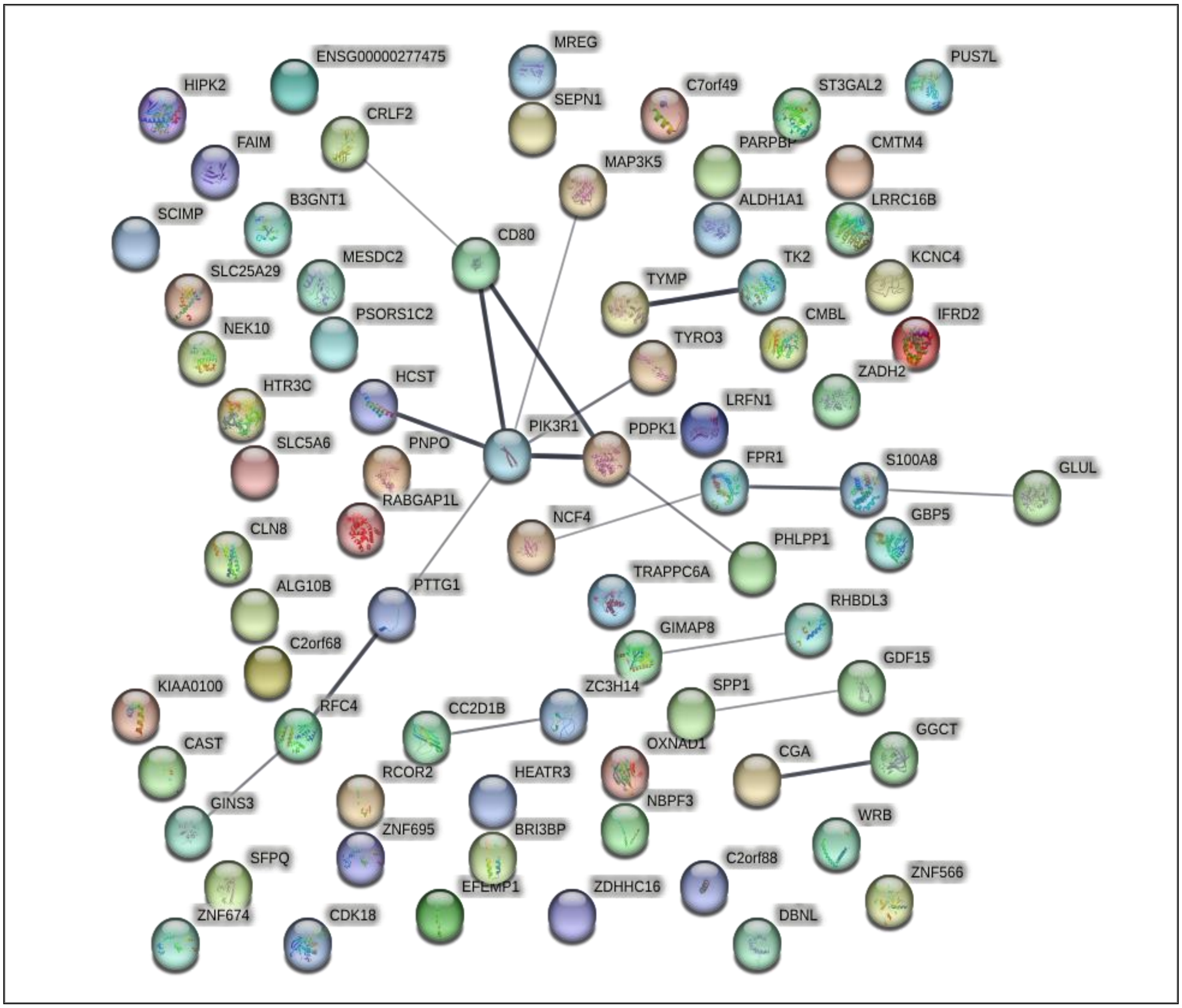

Supplemental Figure 4. Top-ranking networks for differences in gene expression in PHT cells with mTORC2 inhibition (rictor silencing) as compared to control cells. Genes are denoted by gene IDs, green indicates down regulated, red indicates upregulated, gray indicates no difference in gene expression in cells with mTORC2 inhibition as compared to control cells and white denotes the lack of quality signal on the array**.**

ACIN1, apoptotic chromatin condensation inducer 1; AHCTF1, AT hook containing transcription factor 1;AIRE, autoimmune regulator; ARRDC3, arrestin domain containing 3; AURKA, aurora kinase A; C17orf85, chromosome 17 open reading frame 85; C6orf170, chromosome 6 open reading frame 170; CACTIN, cactin, spliceosome C complex subunit; CHD4, chromodomain helicase DNA binding protein 4; CHD6, chromodomain helicase DNA binding protein 6; CHERP, calcium homeostasis endoplasmic reticulum protein; CLDN1, claudin 1; CLK1, CDC-like kinase 1; CYP26A1, cytochrome P450, family 26, subfamily A, polypeptide 1; DENR, density-regulated protein; EIF5, eukaryotic translation initiation factor 5; FAM135A, family with sequence similarity 135, member A; FGD2, FYVE, RhoGEF and PH domain containing 2; HIST2H2BE (includes others), histone cluster 2, H2be; HNRNPA2B1, heterogeneous nuclear ribonucleoprotein A2/B1; HNRNPK, heterogeneous nuclear ribonucleoprotein K; HTRA2, HtrA serine peptidase 2; LSM12, LSM12 homolog (S. cerevisiae); LSM5, LSM5 homolog, U6 small nuclear RNA associated (S. cerevisiae); LST1, leukocyte specific transcript 1; MBD1, methyl-CpG binding domain protein 1; NIN, ninein (GSK3B interacting protein); NUP85, nucleoporin 85kDa; NXF1, nuclear RNA export factor 1; PCBD1, pterin-4 alpha-carbinolamine dehydratase/dimerization cofactor of hepatocyte nuclear factor 1alpha; PIP4K2B, phosphatidylinositol-5-phosphate 4-kinase, type II, beta; PJA2, praja ring finger 2, E3 ubiquitin protein ligase; PPIG, peptidylprolyl isomerase G (cyclophilin G); PTTG1, pituitary tumor-transforming 1; RAE1, RAE1 RNA export 1 homolog (S. pombe); RBCK1, RanBP-type and C3HC4-type zinc finger containing 1; RBM22, RNA binding motif protein 22; RBM4, RNA binding motif protein 4; RNF38, ring finger protein 38; RNF4, ring finger protein 4; RNPS1, RNA binding protein S1, serine-rich domain;RPS12, ribosomal protein S12; RSPRY1, ring finger and SPRY domain containing 1; SCNN1B, sodium channel, non-voltage-gated 1, beta subunit; SF3B4, splicing factor 3b, subunit 4, 49kDa; SNIP1, Smad nuclear interacting protein 1; SNRNP70, small nuclear ribonucleoprotein 70kDa (U1); SNRPD3, small nuclear ribonucleoprotein D3 polypeptide 18kDa; SREK1, splicing regulatory glutamine/lysine-rich protein 1; STK19, serine/threonine kinase 19; TAB3, TGF-beta activated kinase 1/MAP3K7 binding protein 3; TCF20, transcription factor 20 (AR1); TJP2, tight junction protein 2; TRIM23, tripartite motif containing 23; TRIM27, tripartite motif containing 27; TRIM28, tripartite motif containing 28; TRIM5, tripartite motif containing 5; TRIM9, tripartite motif containing 9; UBE2D3, ubiquitin-conjugating enzyme E2D 3; UBR5, ubiquitin protein ligase E3 component n-recognin 5; USP16, ubiquitin specific peptidase 16; USP2, ubiquitin specific peptidase 2; USP21, ubiquitin specific peptidase 21; USP49, ubiquitin specific peptidase 49; USP53, ubiquitin specific peptidase 53; ZDHHC13, zinc finger, DHHC-type containing 13; ZNF567, zinc finger protein 567; Ubiquitin; DUB; snRNP.

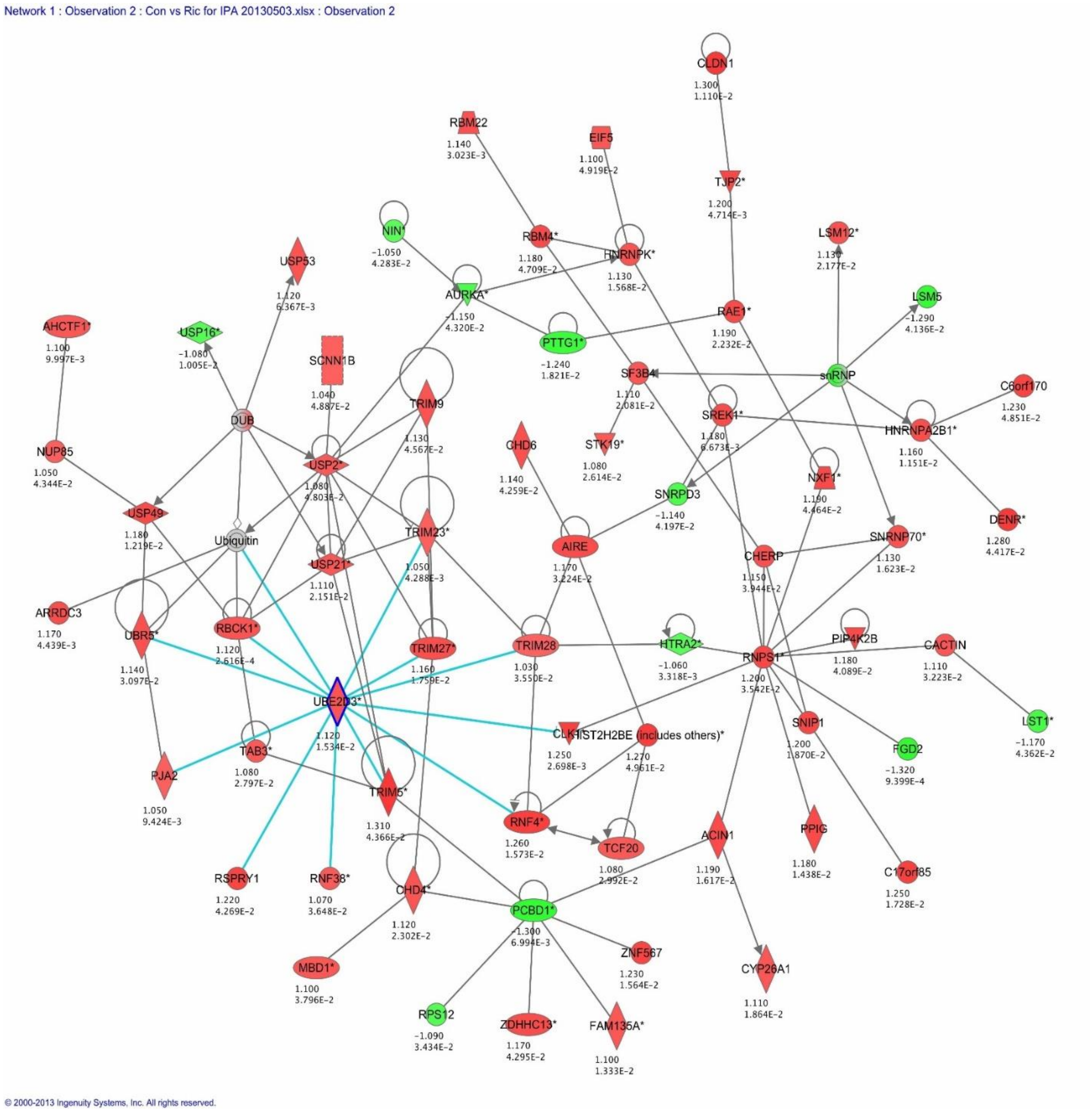

Supplemental Figure 4

Supplemental Figure 5. Top-ranking networks for differences in gene expression in PHT cells with mTORC2 inhibition (rictor silencing) as compared to control cells. Genes are denoted by gene IDs, green indicates down regulated, red indicates upregulated, gray indicates no difference in gene expression in cells with mTORC2 inhibition as compared to control cells and white denotes the lack of quality signal on the array**.**

ALDH1A1,aldehyde dehydrogenase 1 family, member A1; ALDH3B1, aldehyde dehydrogenase 3 family, member B1; ALDH4A1, aldehyde dehydrogenase 4 family, member A1; ALPL, alkaline phosphatase, liver/bone/kidney; ARID4B, AT rich interactive domain 4B (RBP1-like); BAX, BCL2-associated X protein; BECN1, beclin 1, autophagy related; BLZF1, basic leucine zipper nuclear factor 1; CASP9, caspase 9, apoptosis-related cysteine peptidase; COPG2, coatomer protein complex, subunit gamma 2; CSHL1, chorionic somatomammotropin hormone-like 1; DUSP14, dual specificity phosphatase 14; EED, embryonic ectoderm development; EIF4G2, eukaryotic translation initiation factor 4 gamma, 2;EPB41L1, erythrocyte membrane protein band 4.1-like 1; FAM134A, family with sequence similarity 134, member A; FBXO3, F-box protein 3; GPX4 glutathione peroxidase 4; GPX7 glutathione peroxidase 7; GSTK1 glutathione S-transferase kappa 1; HEATR3 HEAT repeat containing 3; IFITM3, interferon induced transmembrane protein 3; IPO13, importin 13; ITPR3, inositol 1,4,5-trisphosphate receptor, type 3; KLF4, Kruppel-like factor 4 (gut); KMT2C, lysine (K)-specific methyltransferase 2C; LXN, latexin; MAML1, mastermind-like 1 (Drosophila); MCL1, myeloid cell leukemia sequence 1 (BCL2-related); NOC4L, nucleolar complex associated 4 homolog (S. cerevisiae); NUDT1, nudix (nucleoside diphosphate linked moiety X)-type motif 1; NUP50, nucleoporin 50kDa; PDCD4, programmed cell death 4 (neoplastic transformation inhibitor); PDK4, pyruvate dehydrogenase kinase, isozyme 4; PHF1, PHD finger protein 1; PHLDA3, pleckstrin homology-like domain, family A, member 3; PPM1A, protein phosphatase, Mg2+/Mn2+ dependent, 1A; PPM1D, protein phosphatase, Mg2+/Mn2+ dependent, 1D; PPP1R12C, protein phosphatase 1, regulatory subunit 12C; PPP1R8, protein phosphatase 1, regulatory subunit 8; PPP2R3A, protein phosphatase 2, regulatory subunit B", alpha; PPP6R3, protein phosphatase 6, regulatory subunit 3; QARS, glutaminyl-tRNA synthetase; RAB3GAP2, RAB3 GTPase activating protein subunit 2 (non-catalytic); Rab5, regulatory factor X-associated ankyrin-containing protein; RBM48, RNA binding motif protein 48; RFXANK, regulatory factor X-associated ankyrin-containing protein; SIRT1, sirtuin 1; SETD8, SET domain containing (lysine methyltransferase) 8; SLC3A2, solute carrier family 3 (activators of dibasic and neutral amino acid transport), member 2; SLC7A10, solute carrier family 7 (neutral amino acid transporter light chain, asc system), member 10; SOST, sclerostin, SPRR2G, small proline-rich protein 2G; TGS1 trimethylguanosine synthase 1; TLE6, transducin-like enhancer of split 6 (E(sp1) homolog, Drosophila); TSC22D1, TSC22 domain family, member 1; ULK1, unc-51 like autophagy activating kinase 1; USO1, USO1 vesicle transport factor; UVRAG, UV radiation resistance associated; ZNHIT6, zinc finger, HIT-type containing 6; Rsk; SWI-SNF; Pp2c; ALDH, glutathione peroxidase; Alp; phosphatase.

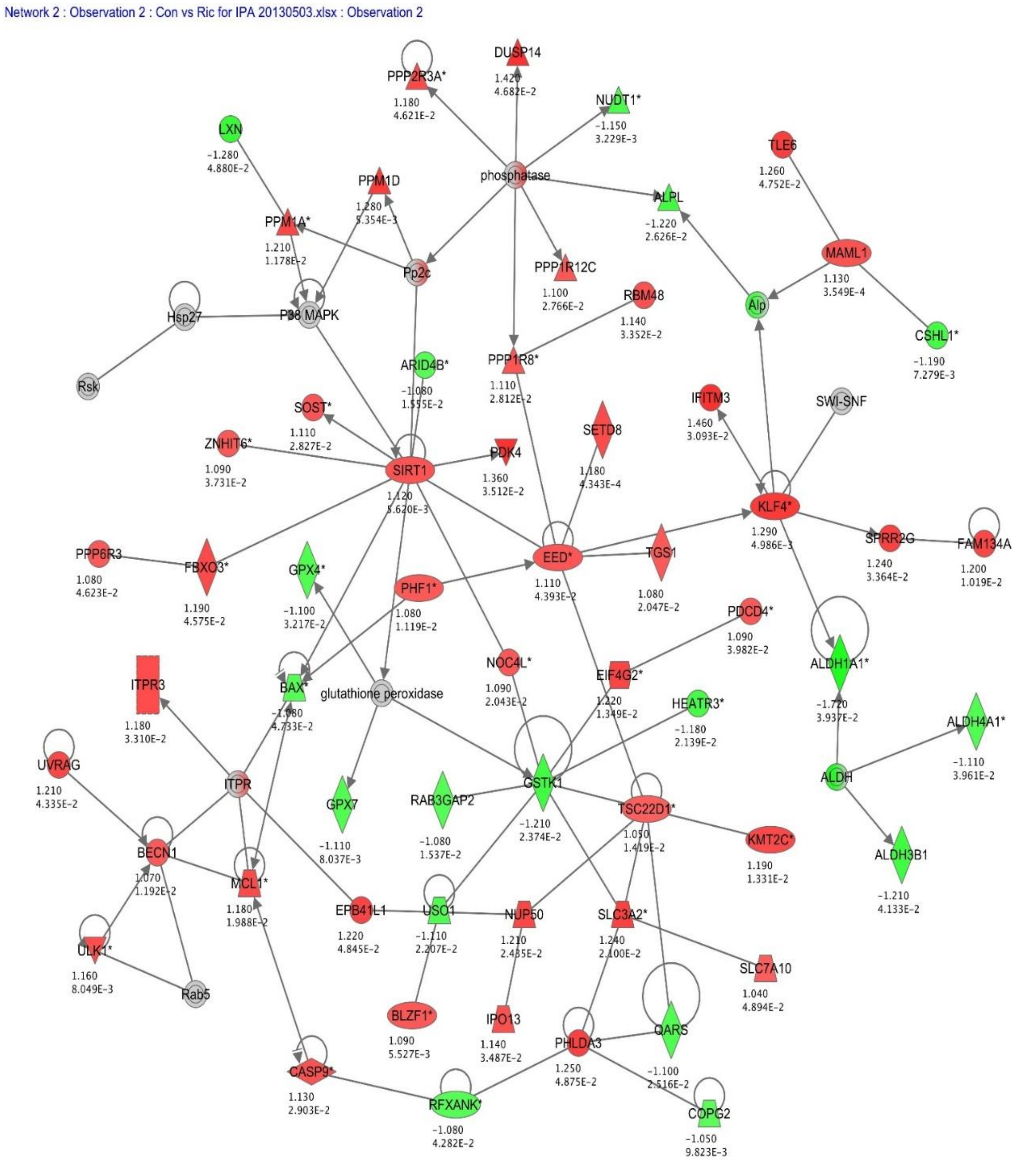

© 2000-2013 Ingenuity Systems, Inc. All rights reserved.

Supplemental Figure 5

Supplemental Figure 6. Top-ranking networks for differences in gene expression in PHT cells with mTORC2 inhibition (rictor silencing) as compared to control cells. Genes are denoted by gene IDs, green indicates down regulated, red indicates upregulated, gray indicates no difference in gene expression in cells with mTORC2 inhibition as compared to control cells and white denotes the lack of quality signal on the array**.**

ABI3, ABI family, member 3; ABLIM1, actin binding LIM protein 1; ANKRD17, ankyrin repeat domain 17; ASB6, ankyrin repeat and SOCS box containing 6; ATP6AP1L, ATPase, H+ transporting, lysosomal accessory protein 1-like; ATP6V0E2, ATPase, H+ transporting V0 subunit e2; CALCOCO2, calcium binding and coiled-coil domain 2; CEACAM1, embryonic antigen-related cell adhesion molecule 1 (biliary glycoprotein); CHORDC1, carcinocysteine and histidine-rich domain (CHORD) containing 1; CRISPLD2, cysteine-rich secretory protein LCCL domain containing 2; CSE1L, CSE1 chromosome segregation 1-like (yeast); CSNK1A1, casein kinase 1, alpha 1; DNAJB1, DnaJ (Hsp40) homolog, subfamily B, member 1; DNAJB12, DnaJ (Hsp40) homolog, subfamily B, member 12; DNAJC13, DnaJ (Hsp40) homolog, subfamily C, member 13; DNAJC28, DnaJ (Hsp40) homolog, subfamily C, member 28; DYRK1A, dual-specificity tyrosine-(Y)-phosphorylation regulated kinase 1A; EEF1A1, eukaryotic translation elongation factor 1 alpha 1; EIF4E2, eukaryotic translation initiation factor 4E family member 2; EIF4ENIF1, eukaryotic translation initiation factor 4E nuclear import factor 1; EZR, ezrin; FAM107A, family with sequence similarity 107, member A; FBXO2, F-box protein 2; GBA, glucosidase, beta, acid; GINS3, GINS complex subunit 3 (Psf3 homolog); HEXB, hexosaminidase B (beta polypeptide); HSCB, HscB iron-sulfur cluster co-chaperone homolog (E. coli); HSPA4, heat shock 70kDa protein 4; IQCB1, IQ motif containing B1; IRAK1, interleukin-1 receptor-associated kinase 1; KBTBD2, kelch repeat and BTB (POZ) domain containing 2; LTN1, listerin E3 ubiquitin protein ligase 1; MARCKS, myristoylated alanine-rich protein kinase C substrate;MRPL16, mitochondrial ribosomal protein L16; MSN, moesin; NCF4, neutrophil cytosolic factor 4, 40kDa; NLRP2, NLR family, pyrin domain containing 2; NPEPPS, aminopeptidase puromycin sensitive; PAQR7, progestin and adipoQ receptor family member VII; PFAS, phosphoribosylformylglycinamidine synthase; PHLPP1, PH domain and leucine rich repeat protein phosphatase 1; PSAP, prosaposin; PSG2, pregnancy specific beta-1-glycoprotein 2; PTGFRN, prostaglandin F2 receptor inhibitor; RIPK2, receptor-interacting serine-threonine kinase 2; RRBP1, ribosome binding protein 1; RSAD2, radical S-adenosyl methionine domain containing 2; SELL, selectin L; SNX10, sorting nexin 10; TNFRSF10B, tumor necrosis factor receptor superfamily, member 10b; TRIM13, tripartite motif containing 13; TXNRD1, thioredoxin reductase 1; TYMP, thymidine phosphorylase; WDR20, WD repeat domain 20; ZNF598, zinc finger protein 598; Tubulin; Vacuolar H+ ATPase; Myosin; Nuclear factor 1; Actin; alcohol group acceptor phosphotransferase; ERK; Erm; estrogen receptor; Calmodulin; CaMKII; HSP; Hsp22/Hsp40/Hsp90; Hsp90; F Actin.

Network 3 : Observation 2 : Con vs Ric for IPA 20130503.xlsx : Observation 2

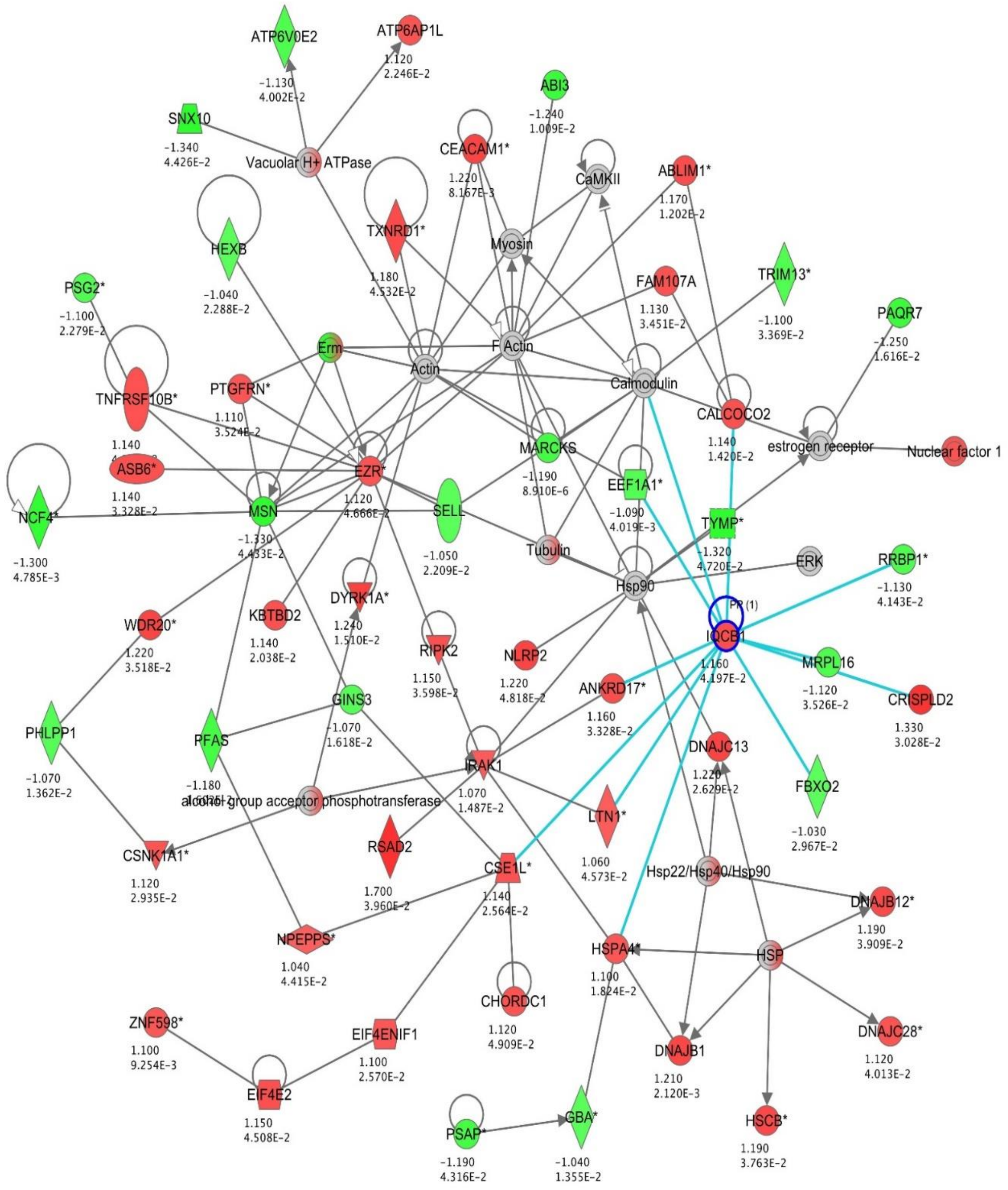

© 2000-2013 Ingenuity Systems, Inc. All rights reserved.

Supplemental Figure 6

Supplemental Figure 7. Correlation between placental mTORC2 functional readouts and the protein expression of osteopontin and sodium-dependent multivitamin transporter (SLC5A6). (A) Representative western blots of osteopontin and sodium-dependent multivitamin transporter (SLC5A6) expression in homogenates of AGA and IUGR placentas. Equal loading was performed. (B, D) Relative expression of osteopontin and SLC5A6 in homogenates of AGA and IUGR placentas. (C, F) Correlation between placental mTORC2 functional readouts AKT$^{Ser\text{-}473}$ and osteopontin and SLC5A6 expression. r= Pearson correlation coefficient, n= AGA, 19; IUGR, 25. Pearson correlation analysis was used to examine the relation among the investigated factors (GraphPad Prism version 5). Osteopontin, AGA, r=0.6148, p= 0.0001; IUGR r=0.5589, p= 0.0001. SLC5A6, AGA, r=0.8731, p= 0.0001; IUGR r=0.8619, p= 0.0001.

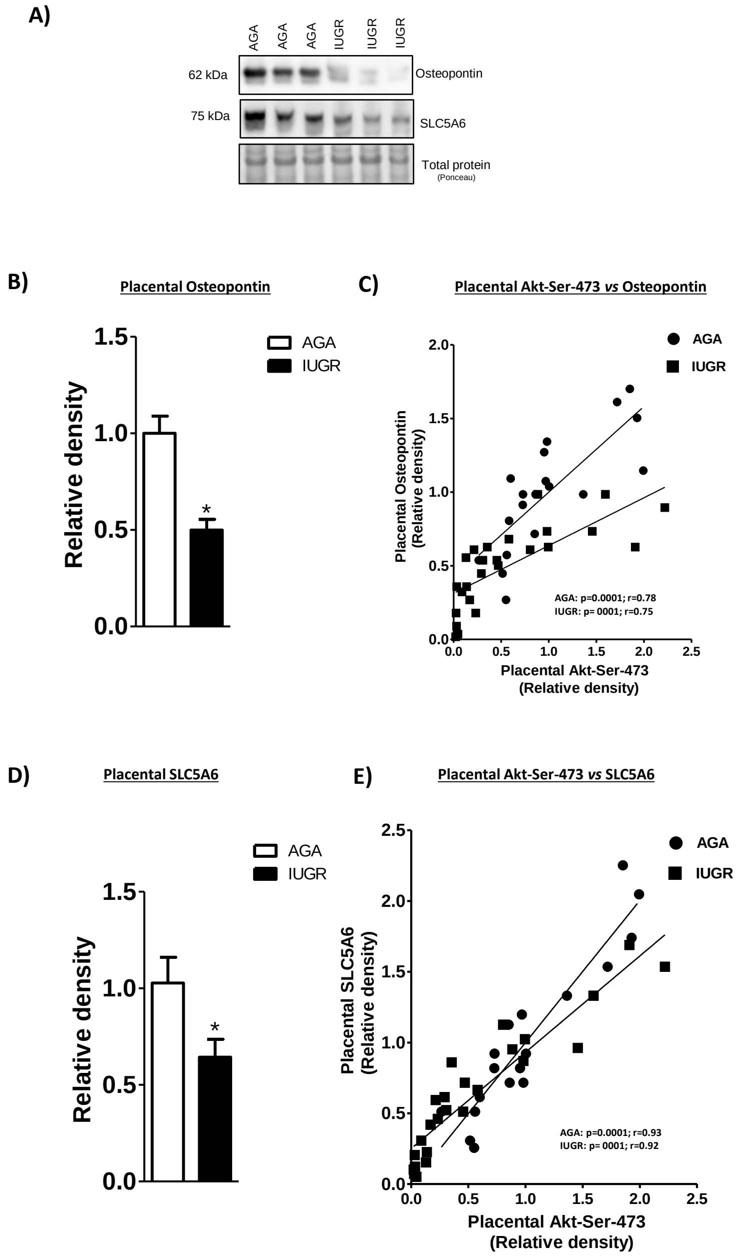

Supplemental Figure 8: Network analysis of down-regulated (pseudo) genes in rictor silenced (mTORC2 inhibited) PHT cells. The ShinyGO application (version 0.66) (9) was used for exploring enrichment in Network analysis using the 30 top genes obtained from the gene-based association analyses. Multiple testing correction was applied using the Benjamini–Hochberg method implemented in the application. We considered significant those processes with false discovery rate (FDR) p-value < 0.05.

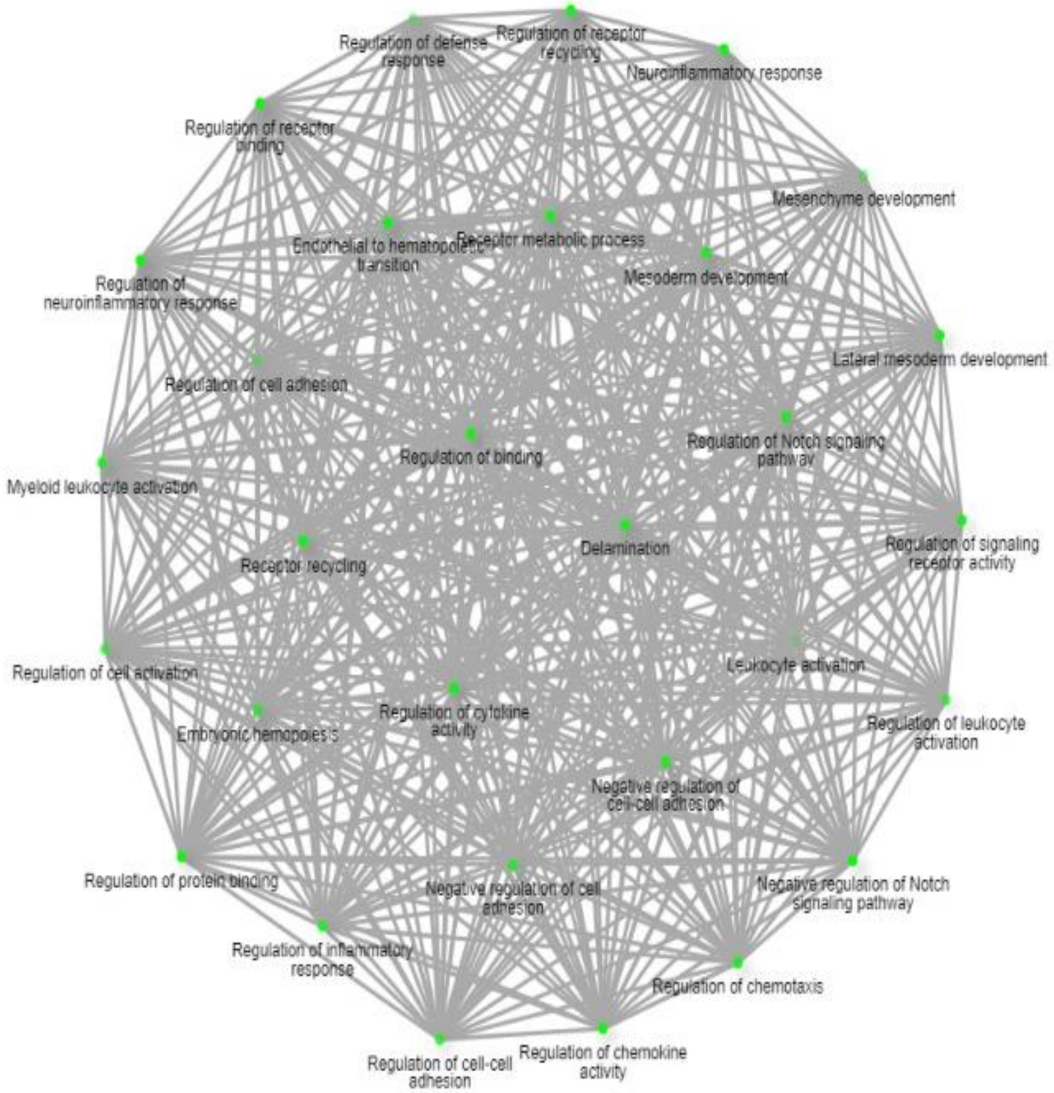

References

1. Bayerlova, M., Jung, K., Kramer, F., Klemm, F., Bleckmann, A., and Beissbarth, T. (2015) Comparative study on gene set and pathway topology-based enrichment methods. *BMC Bioinformatics* **16**, 334
2. Kanehisa, M., Furumichi, M., Tanabe, M., Sato, Y., and Morishima, K. (2017) KEGG: new perspectives on genomes, pathways, diseases and drugs. *Nucleic Acids Res* **45**, D353-D361
3. Doniger, S. W., Salomonis, N., Dahlquist, K. D., Vranizan, K., Lawlor, S. C., and Conklin, B. R. (2003) MAPPFinder: using Gene Ontology and GenMAPP to create a global gene-expression profile from microarray data. *Genome Biol* **4**, R7
4. Ogata, H., Goto, S., Sato, K., Fujibuchi, W., Bono, H., and Kanehisa, M. (1999) KEGG: Kyoto Encyclopedia of Genes and Genomes. *Nucleic Acids Res* **27**, 29-34
5. Kanehisa, M., and Goto, S. (2000) KEGG: kyoto encyclopedia of genes and genomes. *Nucleic Acids Res* **28**, 27-30
6. Kanehisa, M., Sato, Y., Kawashima, M., Furumichi, M., and Tanabe, M. (2016) KEGG as a reference resource for gene and protein annotation. *Nucleic Acids Res* **44**, D457-462
7. Szklarczyk, D., Morris, J. H., Cook, H., Kuhn, M., Wyder, S., Simonovic, M., Santos, A., Doncheva, N. T., Roth, A., Bork, P., Jensen, L. J., and von Mering, C. (2017) The STRING database in 2017: quality-controlled protein-protein association networks, made broadly accessible. *Nucleic Acids Res* **45**, D362-D368
8. Rosario, F. J., Kanai, Y., Powell, T. L., and Jansson, T. (2013) Mammalian target of rapamycin signalling modulates amino acid uptake by regulating transporter cell surface abundance in primary human trophoblast cells. *J Physiol* **591**, 609-625
9. Ge, S. X., Jung, D., and Yao, R. (2020) ShinyGO: a graphical gene-set enrichment tool for animals and plants. *Bioinformatics* **36**, 2628-2629
